# Achieving 27.7% Efficiency with a Mechanically Stacked, Four‐Terminal Perovskite/InGaAsP Tandem Solar Cell

**DOI:** 10.1002/smsc.70298

**Published:** 2026-05-09

**Authors:** Bikesh Gupta, The Duong, Tuomas Haggren, Daniel Walter, Julie Tournet, Azul Osorio Mayon, Chennupati Jagadish, Hark Hoe Tan, Siva Karuturi

**Affiliations:** ^1^ Department of Electronic Materials Engineering Research School of Physics The Australian National University Canberra Australia; ^2^ School of Engineering The Australian National University Canberra Australia; ^3^ ARC Centre of Excellence for Transformative Meta‐Optical Systems Research School of Physics The Australian National University Canberra Australia

**Keywords:** carrier‐selective contact, InGaAsP, passivation, perovskite, solar cells, tandem

## Abstract

Multijunction solar cell architectures offer a means to exceed the efficiency limits of traditional single‐junction cells, notably surpassing the Shockley–Queisser limit. III–V compound semiconductors, known for their adaptable bandgaps, are often used in constructing multijunction cells, but their fabrication largely relies on complex epitaxial growth techniques. These processes are further complicated by the demanding necessity for lattice‐matched, heavily‐doped tunnel junctions. To address these challenges, our study introduces a mechanically stacked, four‐terminal perovskite/InGaAsP tandem solar cell as a viable alternative to conventional all‐III–V semiconductor dual‐junction cells. We successfully achieved a low‐bandgap InGaAsP solar cell with an impressive efficiency of 19.0% and an open‐circuit voltage of 657 mV by employing carrier‐selective contacts, a performance that rivals state‐of‐the‐art InGaAsP homojunction solar cells. Furthermore, by pairing an InGaAsP bottom cell with a semi‐transparent perovskite top cell in a tandem configuration, we attained a remarkable efficiency of 27.7% along with an outstanding open‐circuit voltage of 1.7 V. The remarkable proof‐of‐concept demonstration presented here not only paves the way for highly efficient dual‐junction thin film flexible solar cells but also simplifies the fabrication process by eliminating the need for lattice‐matched tunnel junction layers, a common requirement in conventional III–V multijunction solar cells.

## Graphical abstract

A low‐bandgap InGaAsP heterojunction solar cell is demonstrated, achieving 19% efficiency via interface‐engineered InP passivation and TiO_2_ electron‐selective contact. Integrated into a mechanically stacked perovskite/III–V tandem, it delivers 27.7% efficiency. Loss analysis reveals clear pathways toward >30% tandem performance, highlighting a versatile platform for high‐efficiency, bandgap‐engineered hybrid photovoltaics.

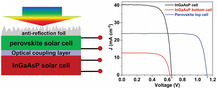

## 1 Introduction

The quest for carbon‐free energy solutions has become increasingly critical in the face of increasing global energy demands and environmental concerns. Among myriad renewable energy sources, solar energy stands out for the ubiquity of the solar resource. The earth receives an abundant supply of solar irradiation, with the potential to produce significantly more energy than current global consumption rates [1]. Photovoltaic (PV) technology, which converts sunlight directly into electricity via the PV effect, has been at the forefront of harnessing solar energy. Over the years, significant advancements in PV technology have led to considerable improvements in solar cell efficiency and a reduction in manufacturing costs, making solar energy more accessible and affordable. Among the various materials and technologies explored for PV applications, two distinct classes of materials have garnered substantial attention due to their promising characteristics: III–V compound semiconductors and hybrid organic–inorganic perovskite semiconductors [2, 3].

III–V compound semiconductors have been extensively studied for their superior optoelectronic properties [4]. These materials boast a direct bandgap, enabling efficient absorption of solar irradiation even in thin layers, a critical attribute for high‐efficiency solar cells [5]. The ability to fine‐tune the bandgap of III–V compounds by varying their compositional ratios offers a versatile tool for optimizing solar cell performance across different spectral regions. This tunability is particularly advantageous in tandem solar cell configurations, where several III–V semiconductor layers with different bandgaps are stacked to harness a broader spectrum of solar radiation, thereby surpassing the efficiency limits of traditional single‐junction solar cells. Despite their promising characteristics, the widespread adoption of III–V semiconductors in solar cell applications has been hampered by several challenges. The fabrication of III–V‐based solar cells predominantly relies on sophisticated epitaxial growth techniques which are complex and costly [6]. Additionally, constructing multijunction solar cells from III–V materials necessitates the formation of lattice‐matched, heavily‐doped tunnel junctions to facilitate efficient carrier transport between the subcells, adding another layer of complexity to the fabrication process [7, 8].

Along with III–V semiconductors, hybrid organic–inorganic perovskite semiconductors have emerged as a disruptive technology in the field of PVs due to their remarkable optoelectronic properties and the simplicity of their fabrication processes [9]. Perovskite solar cells exhibit strong absorption in the visible spectrum, enabling them to convert a significant portion of sunlight into electricity. The tunable bandgap of perovskites, similar to III–V semiconductors, provides flexibility in device engineering and optimization for various applications [10]. Other advantageous properties include long carrier diffusion lengths, low exciton binding energies, and high carrier mobilities, all of which contribute to high device efficiencies [11, 12]. One of the most compelling aspects of perovskite solar cells is their compatibility with solution‐based processing techniques, which significantly simplifies the manufacturing process and reduces costs compared to traditional solar cell fabrication methods [13]. The ability to produce perovskite solar cells as thin, lightweight, and flexible films further expands their applicability, enabling integration into a wide array of surfaces and devices. Since their inception, the efficiencies of perovskite solar cells have seen an unprecedented rise, reaching over 25% in just a few years, making them one of the fastest‐advancing solar technologies to date [14].

The integration of perovskite solar cells with other semiconductor materials in tandem configurations has attracted considerable research interest as a strategy to overcome the single‐junction Shockley–Queisser efficiency limit [15, 16]. Tandem solar cells utilize multiple layers of semiconductors, each with a distinct bandgap, to better match the solar spectrum and reduce thermalization and transmission losses [17]. Among various tandem combinations, perovskite/silicon tandems have been extensively studied, benefiting from the maturity and stability of silicon technology [18]. However, the suboptimal bandgap of silicon and the thickness required for effective light absorption, due to its indirect bandgap nature, pose limitations to the efficiency and flexibility of such tandem cells [19, 20]. Perovskite/CI(G)S tandem solar cells are another material combination that has been extensively researched [21].

Beyond silicon‐based tandems, III–V semiconductors have long been recognized as ideal candidates for high‐efficiency multijunction solar cells due to their direct bandgaps and superior optoelectronic properties. Conventional III–V tandems, such as GaInP/GaAs, are typically realized through epitaxial growth and have achieved outstanding performance, albeit at high cost and limited scalability. More recently, perovskite top cells have been explored in combination with III–V absorbers (primarily through simulations), offering a potential pathway toward high‐efficiency hybrid tandems [22, 23]. However, in contrast to the extensive literature on perovskite/silicon tandems, reports on such perovskite/III–V systems remain limited.

In this study, we report an innovative perovskite/InGaAsP tandem solar cell featuring a four‐terminal configuration that showcases exceptional efficiency. The bandgap of InGaAsP can be precisely tuned from 0.73 to 1.35 eV when lattice‐matched to InP, making it an attractive bottom cell in tandem configurations [24]. Here, we develop a narrow‐bandgap InGaAsP solar cell, utilizing a TiO_2_ electron‐selective contact layer, achieving an outstanding efficiency of 19.0% alongside an open‐circuit voltage of 657 mV. This remarkable performance not only exceeds the efficiency of ∼10% achieved previously for heterojunction architectures but also rivals the efficiencies of conventional InGaAsP homojunction solar cells [25, 26, 27]. Moreover, solar cell architectures employing carrier selective contacts offer a streamlined fabrication process for PV devices in contrast to epitaxially grown homojunction counterparts and thus enhancing the economic viability. Furthermore, we present a mechanically stacked tandem solar cell structure, combining the InGaAsP bottom cell with a semi‐transparent perovskite (*E*_*g*_ = 1.55 eV) top cell. This proof‐of‐concept tandem architecture demonstrates a remarkable efficiency of 27.7%, coupled with a superior open‐circuit voltage of 1.7 V, a significant achievement in tandem solar cell technology. By integrating a semi‐transparent perovskite top cell with a low‐bandgap InGaAsP bottom cell, we address both the challenge of spectral mismatch and the need for cost‐effective, flexible solar solutions.

## 2 Results and Discussion

### 2.1 InGaAsP Growth and Characterization

**FIGURE 1:**
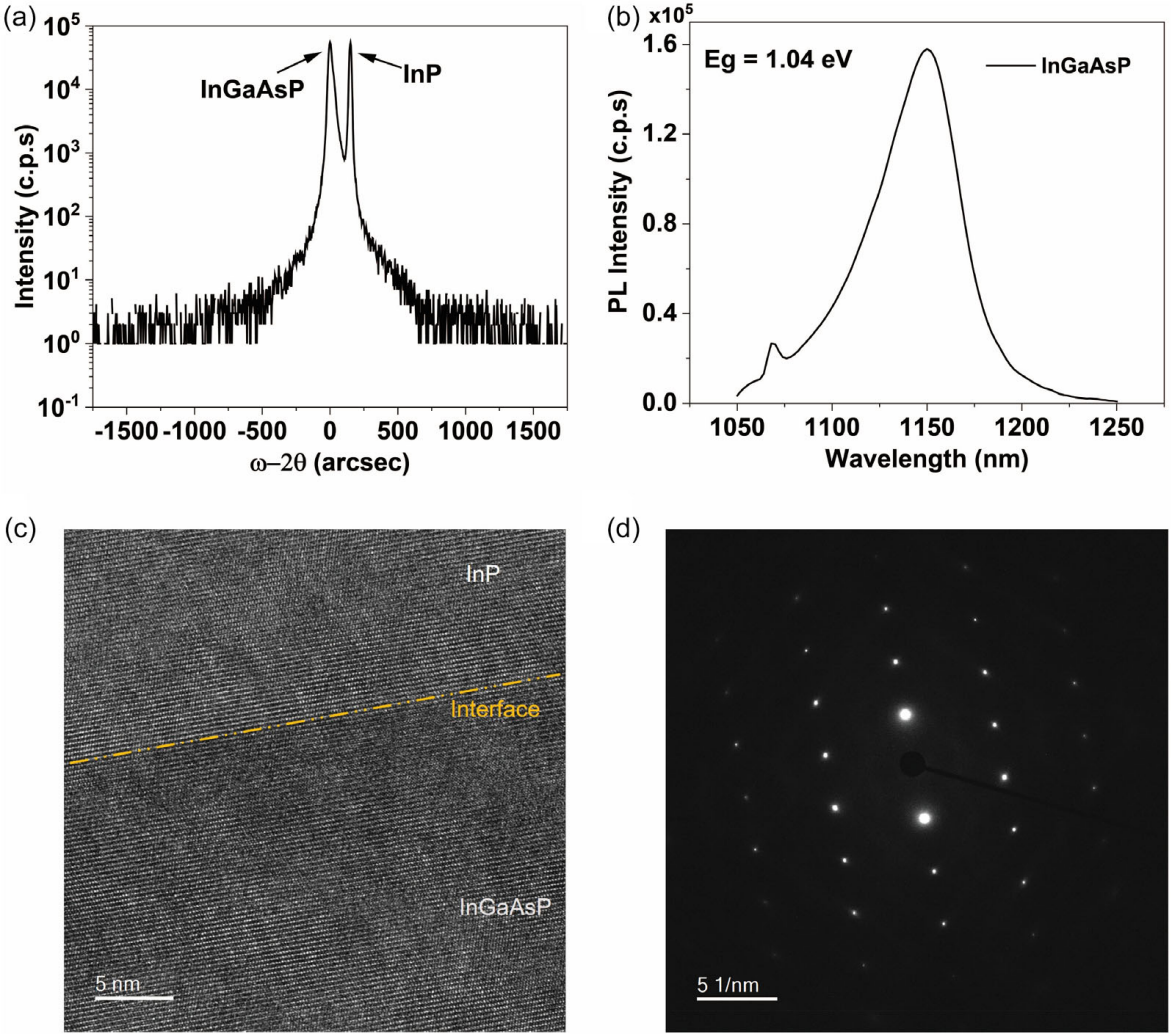
InGaAsP material characterization. (a) *ω–2θ* (004) high‐resolution X‐ray diffraction scan, (b) photoluminescence spectrum, (c) high‐resolution transmission electron microscopy image, and (d) selected area electron diffraction of InGaAsP grown on InP substrate.

Ensuring a high‐quality epitaxial growth of the quaternary InGaAsP alloy is crucial, as it directly impacts the performance of PV devices. The quality of the epitaxial growth for a 1.5 µm‐thick InGaAsP layer is investigated using various techniques, as depicted in Figure 1. The composition of the InGaAsP alloy is determined through a combination of high‐resolution X‐ray diffraction (HRXRD) and photoluminescence (PL). In Figure 1a, the *ω–2θ* plot of the InGaAsP alloy reveals a determined lattice constant of 5.89 Å. The angular peak splitting between the InGaAsP epilayer and the InP substrate is measured at only −151 arcsec, indicating a nominal mismatch of 8.0 × 10^−4^. This value is commonly acknowledged in the literature as a lattice‐matched condition for the thickness considered [28]. To distinguish between the layer and substrate peaks, an initial scan was conducted, followed by a second scan after chemically etching the top layer (quaternary material) by a few hundred nanometers. The reduction in peak intensity enabled us to identify the left peak as corresponding to the layer. The *ω–2θ* scan also indicates the absence of significant phase decomposition in the alloy, displaying a well‐defined and intense peak. The full width at half maximum (FWHM) of the InGaAsP layer measures 29 arcsec, while the FWHM of the substrate is 14 *arcsec*, a level comparable to the best values reported in the literature [29]. PL spectroscopy is employed to assess both the optical quality and bandgap of the InGaAsP alloy. As depicted in Figure 1b, the PL spectra exhibit an exceptionally high intensity, indicating occurrence of radiative recombination within the InGaAsP bulk. This observation suggests that the epitaxial growth process has yielded a high‐quality material, characterized by a reduced presence of non‐radiative defective centers in the bulk of InGaAsP. This quality improvement is crucial for the development of high‐performance optical devices [30]. The peak emission at the band edge of InGaAsP, as observed in the PL spectra, is approximately 1192 nm (*E*_*g*_ = 1.04 eV). Utilizing the lattice constant of 5.89 Å and the bandgap of 1.04 eV, the composition of InGaAsP is determined through fitting the *ω–2θ* curve using the model outlined by Adachi et al. [4] The derived composition is In_0.79_Ga_0.21_As_0.44_P_0.56_.

To further analyze the variation in composition within the InGaAsP layer and at the interface between the InGaAsP epilayer and InP substrate, high‐resolution transmission electron microscopy (HRTEM) was conducted on different sections of the layer, as illustrated in Figures 1c and S1. The bright and dark field micrographs of the InGaAsP layer, presented in Figure S1, reveal consistent contrast, indicating uniform composition among all components. This observation suggests the absence of spinodal‐like decomposition into binary or ternary compounds, a significant finding given the susceptibility of InGaAsP quaternary alloys to compositional fluctuations due to the miscibility gap [31, 32]. Moreover, the well‐defined interface between InP and the InGaAsP epilayer, as depicted in Figure 1c, exhibits nearly perfect lattice matching, with no observable signs of defects [33]. Effective carrier transport across the interface in the devices relies on precise lattice matching, as an interface with lattice mismatch can introduce defects that serve as centers for carrier recombination [34]. Additionally, the sharp selected area electron diffraction patterns (Figure 1d) confirm the high quality of InGaAsP epilayers suitable for optical applications.

### 2.2 Photovoltaic Performance Evaluation

**FIGURE 2:**
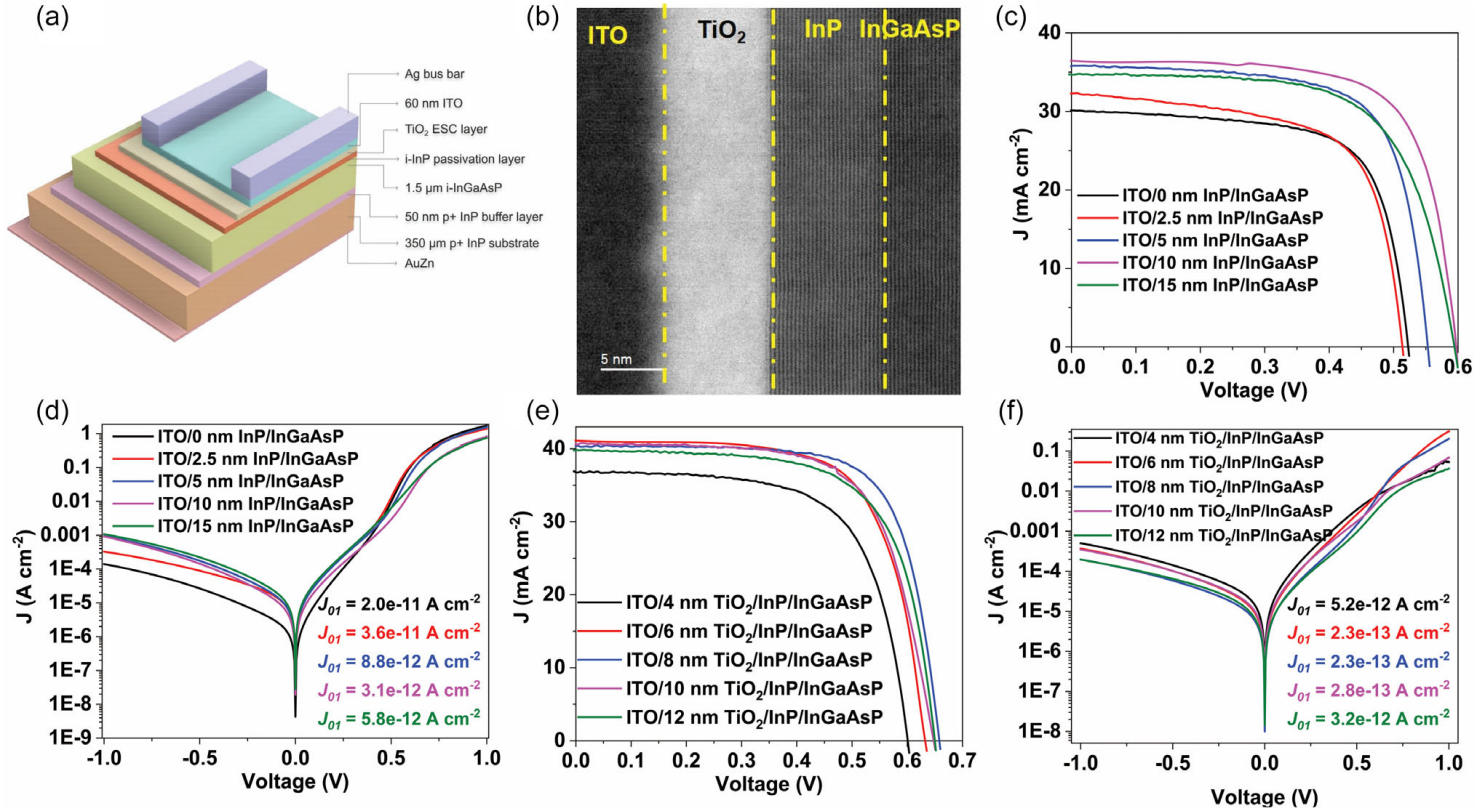
InGaAsP photovoltaic performance evaluation. (a) Schematic representation of InGaAsP solar cell depicting different layers in the structure. (b) Cross‐sectional high‐resolution transmission electron microscopy of the InGaAsP solar cell. (c) Light *J–V* curve and (d) dark *J–V* curve of InGaAsP solar cell with different thickness of InP passivation layer. (e) Light *J–V* curve and (f) dark *J–V* curve of InGaAsP solar cell with different thickness of TiO_2_ electron selective contact layer with 10 nm‐thick InP passivation layer. The inset in the dark *J–V* curves shows the *J*_01_ component of the dark current obtained by fitting the dark current using double diode model.

InGaAsP heterojunction solar cells were fabricated employing TiO_2_ electron‐selective contact (ESC) layer. The overall process for fabricating the solar cells is illustrated in Figure S2. In a summary, the solar cells were fabricated by first depositing a TiO_2_ ESC layer, followed by an indium tin oxide (ITO) transparent contact oxide, and then Ag bus bars. The schematic representation of the InGaAsP heterojunction solar cell structure is shown in Figure 2a. A notable characteristic of this device structure is the epitaxial growth of only InGaAsP and an ultrathin surface passivation layer. Consequently, the film lacks doping, back‐surface field layers, or window layers commonly found in high‐efficiency solar cells, leading to a substantial reduction in device complexity and fabrication steps [35]. The top surface of the device was examined using HRTEM, and the associated micrograph is displayed in Figure 2b. In this micrograph, the distinct interfaces of ITO, an 8 nm‐thick TiO_2_ ESC layer, and a 10 nm‐thick InP passivation layer on top of InGaAsP are clearly visible. The micrograph indicates that the amorphous nature of the TiO_2_ ESC layer, while InP is perfectly lattice matched with InGaAsP, showing no interface imperfections. Additionally, elemental mapping of the corresponding micrograph reveals well‐defined interfaces between each layer, with no inter‐diffusion of elements in each layer (Figure S3). The InP layer in this structure plays a crucial role in passivating the InGaAsP surface.

Achieving effective passivation of III–V semiconductors is crucial for attaining high PV performance, particularly in terms of achieving a high photovoltage [36, 37]. In this case, we selected undoped InP as the passivation layer due to its outstanding lattice matching with low‐bandgap InGaAsP. Additionally, it inherently exhibits a lower surface recombination velocity compared to other III–V semiconductors [38, 39]. Furthermore, InP has been shown to decrease the surface recombination velocity at the interface with InGaAsP [40]. In our current investigation, we also noted that a thin InP layer could amplify the PL intensity of the InGaAsP layer by nearly 1.5 times, highlighting the effectiveness of InP as a passivation layer for InGaAsP (Figure S4). Determining the optimal thickness for the passivation layer is pivotal in achieving high‐efficiency devices. Thicker layers have demonstrated improved passivation, whereas thinner layers have shown enhanced carrier transport [41]. To assess the optimal thickness of the InP passivation layer, solar cells were fabricated with varying InP thicknesses (2.5, 5, 10, and 15 nm) and without any TiO_2_ ESC layer. The light current–voltage (*J–V*) characteristics of each device are depicted in Figure 2c, and the PV parameters are presented in Table 1.

**TABLE 1:** Summary of the performance parameters of various InGaAsP solar cells with different thicknesses of InP passivation layer.

Device	***V***_**oc**_ **(mV)**	***J***_**sc**_ **(mA cm** ^**−2**^ **)**	*FF* (%)	*η* (%)
ITO/InGaAsP	522	30.1	68.8	10.8
ITO/2.5 nm InP/InGaAsP	513	32.3	65.1	10.8
ITO/5 nm InP/InGaAsP	552	35.8	70.2	13.8
ITO/10 nm InP/InGaAsP	596	36.4	70.8	15.3
ITO/15 nm InP/InGaAsP	593	34.7	66.8	13.6

The reference cell lacking an InP layer (i.e., ITO/InGaAsP) exhibited an open‐circuit voltage (*V*_oc_) of 522 mV and a short‐circuit current density (*J*_sc_) of 30.1 mA cm^−2^. With a fill factor (*FF*) of 68.8%, the reference cell displayed a reasonable efficiency of 10.8%. While ITO serves the purpose of a transparent conducting oxide, it has also demonstrated reasonable efficiencies in other III–V semiconductor solar cells as well, thanks to its n‐type nature facilitating photogenerated carrier separation and transport [42, 43]. The efficiency reported in the current cell is comparable to previously reported efficiencies for ITO/InGaAsP solar cells, even though, in those cases, InGaAsP had a bandgap greater than 1.5 eV [44, 45]. However, the *V*_oc_ attained in the current device is at least 100 mV higher than the previously reported value for ITO/InGaAsP solar cells with a similar bandgap [24]. This underscores the superior material quality (lower infterfacial defects and phase decomposition) and optoelectronic properties of the current InGaAsP material compared to the previously reported material [24]. Following the introduction of a 2.5 nm‐thick InP passivation layer, no significant enhancement in device performance was observed, possibly attributed to the nonuniform growth of the thin InP layer. However, with an increased InP thickness to 5 nm, a notable improvement in the *V*_oc_ to 552 mV was recorded. Furthermore, the *J*_sc_ increased to 35.8 mA cm^−2^, the *FF* to 70.2%, resulting in an efficiency of 13.8%. A further increase in InP thickness to 10 nm continued to improve the *V*_oc_ (596 mV), resulting in an efficiency of 15.3%, with *J*_sc_ at 36.4 mA cm^−2^ and *FF* at 70.8%.

The consistent enhancement in *V*_oc_ indicates the effective suppression of surface recombination by the presence of the InP passivation layer [46]. The enhancement in the *J*_sc_ could be attributed to the improved carrier collection facilitated by the presence of the InP layer, as illustrated by the external quantum efficiency (EQE) measurements in Figure S5. Notably, the *V*_oc_ tends to reach a saturation point with a 15 nm‐thick InP layer, while other PV parameters were adversely affected with increased thickness. The *J*_sc_ decreased to 34.7 mA cm^−2^, and the *FF* dropped to 66.8%, resulting in an efficiency of 13.4%. The decline in *J*_sc_ and *FF* could be attributed to the increased resistance to photogenerated carrier transport introduced by the thicker InP layer. This indicates that 10 nm is the optimum thickness for the InP passivation layer in this device, striking a balance between carrier transport and surface passivation [47].

To provide additional insights into the effectiveness of InP as a passivation layer, dark current measurements were conducted on these cells. Dark current measurements offer valuable information about recombination currents in the cell, aiding in the quantification of *V*_oc_ losses. The dark current–voltage (*J–V*) characteristics of the cells were recorded under ambient conditions at 25°C, as depicted in Figure 2d. These dark *J–V* curves were then fitted using a double‐diode model equation [48].

(1) $$J=\;J_{01}\exp\;\frac{\left[q(V-JR_{s})\right]}{n_{1}kT}+\;J_{02}\exp\;\frac{\left[q(V-JR_{s})\right]}{n_{2}kT}+\;\frac{V-JR_{s}}{R_{Sh}}$$

In the equation, *J*_01_ denotes for the recombination current density in the quasi‐neutral bulk region, while *J*_02_ represents the Shockley–Read–Hall (SRH) recombination current density in the space charge regions. The parameters *n*_1_ and *n*_2_ correspond to the ideality factors of the diodes, and *R*_*s*_ and *R*_*sh*_ denote the series and shunt resistances, respectively.

The inset in Figure 2d displays the extracted *J*_01_ component of the dark current. The *J*_01_ value for the ITO/InGaAsP reference cell is 2.0 × 10^−11^ A cm^−2^, whereas the *J*_01_ value for the ITO/InP/InGaAsP cell, after the introduction of a 2.5 nm‐thick InP layer, did not show a significant reduction, remaining at 3.6 × 10^−11^ A cm^−2^. This suggests that 2.5 nm may not be sufficient to effectively passivate the InGaAsP surface, consistent with the observed lack of significant changes in PV performances. However, as the thickness of the InP layer is increased to 5 nm, the *J*_01_ value (8.8 × 10^−12^ A cm^−2^) decreases by one order of magnitude. This reduction further continues (3.1 × 10^−12^ A cm^−2^) when the thickness reaches 10 nm and saturates (5.8 × 10^−12^ A cm^−2^) with a 15 nm‐thick layer. The dark *J–V* characteristics indicate that a 10 nm‐thick InP layer is optimal for InGaAsP passivation. Additionally, a lower *J*_01_ value is indicative of a higher *V*_oc_ based on the following equation [49].

(2) $$V_{\mathrm{oc}}\approx \frac{\;nkT}{q}\;\ln\;\left(\frac{J_{\mathrm{sc}}}{J_{01}}\right)$$

In the equation, *n* represents the diode ideality factor, *k* is the Boltzmann constant, *T* is the absolute temperature of the cell, and *q* denotes the elementary charge of electron.

The consistent reduction in the *J*_01_ value of the dark current, correlated with the increasing thickness of the InP layer, offers a straightforward explanation, as indicated by the above equation, for the improvement observed in the solar cell *V*_oc_. Additionally, *V*_oc_ calculated from Equation (2) closely aligns with the values obtained from light *J–V* measurements, as detailed in Table S2.

After determining the optimal thickness of the InP passivation layer, we proceeded to fabricate devices with a TiO_2_ ESC layer to facilitate the selective transport of electrons from the InGaAsP layer. Various devices were fabricated with different thicknesses of TiO_2_, while maintaining the InP layer at 10 nm. The light *J–V* characteristics are presented in Figure 2e, and the corresponding PV performance parameters are tabulated in Table 2. The introduction of a 4 nm‐thick TiO_2_ layer on top of InP/InGaAsP resulted in solar cell performance similar to that of only a 10 nm‐thick InP layer, suggesting it is insufficient to induce significant band bending at the interface for efficient electron selectivity and transport [46]. Nevertheless, with an increase in the thickness of the TiO_2_ ESC layer, the PV performance improves, reaching an optimal value with an 8 nm‐thick layer with a *V*_oc_ of 657 mV, *J*_sc_ of 40.4 mA cm^−2^, *FF* of 71.8%, and an outstanding efficiency of 19%.

**TABLE 2:** Summary of the performance parameters of various InGaAsP solar cells with different thicknesses of TiO_2_ ESC with 10 nm‐thick InP passivation layer.

Device	***V***_**oc**_ **(mV)**	***J***_**sc**_ **(mA cm** ^**−2**^ **)**	*FF* (%)	*η* (%)
ITO/4 nm TiO_2_/InP/InGaAsP	599	35.5	66.2	14.1
ITO/6 nm TiO_2_/InP/InGaAsP	632	41.0	68.1	17.6
ITO/8 nm TiO_2_/InP/ InGaAsP	657	40.4	71.8	19.0
ITO/10 nm TiO_2_/InP/ InGaAsP	647	40.6	67.5	17.7
ITO/12 nm TiO_2_/InP/ InGaAsP	653	39.3	67.6	17.4

It is noteworthy that the *V*_oc_ and efficiency achieved in this simple heterojunction architecture solar cell rivals those obtained from epitaxially grown state‐of‐the‐art InGaAsP homojunction solar cells with a similar bandgap, and in many cases, outperform most of the reported values in the literature (Table S3) [50]. Furthermore, the solar cells incorporating an 8 nm‐thick TiO_2_ layer in conjunction with a 10 nm InP layer exhibited a remarkably low bandgap‐voltage offset (*W*_oc_ = *E*_*g/q*_‐*V*_oc_) of only 383 mV, closely approaching its theoretical value predicted using a diffusion‐limited model [50]. Remarkably, this value is considerably less than the values documented in the literature [51, 52, 53]. *W*_oc_ is commonly employed to assess the quality of the light‐absorbing material and the junction [54]. The reduced *W*_oc_ value in the current device indicates the superior quality of the InGaAsP material and the formation of an excellent heterojunction facilitated by the TiO_2_ ESC layer. Nevertheless, as the thickness of the TiO_2_ ESC layer is increased further, the PV performance begins to decrease due to the increased resistance introduced by the thicker TiO_2_ ESC layer. This suggests that 8 nm is the optimal thickness for the TiO_2_ ESC layer in the present device.

To investigate the recombination of photogenerated carriers in the presence of the TiO_2_ layer, the dark current of the devices was measured and fitted using a double diode model, as described earlier. The dark *J–V* characteristics are illustrated in Figure 2f, with the *J*_01_ value of the dark current shown in the inset. With the introduction of the TiO_2_ ESC contact layer, *J*_01_ decreases significantly compared to only the InP layer and systematically decreases with increasing thickness, reaching the lowest value of 2.3 × 10^−13^ A cm^−2^ for an 8 nm‐thick TiO_2_ layer. The reduction of *J*_01_ in the presence of TiO_2_ can be attributed to the efficient electron‐selective and hole‐blocking properties of the TiO_2_ layer, which decrease the probability of recombination at the interface. The decrease in *J*_01_ provides a clear explanation for the substantial improvement in the solar cell *V*_oc_ in the presence of the TiO_2_ ESC layer.

**FIGURE 3:**
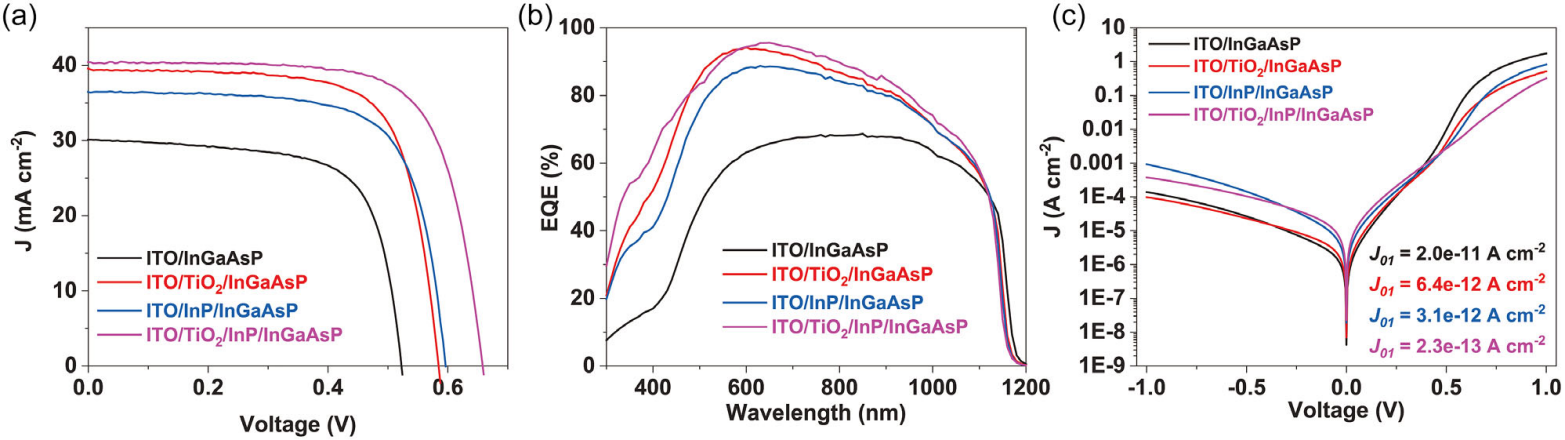
(a) Light *J–V* curve, (b) external quantum efficiency, and (c) dark *J–V* curve of InGaAsP solar cell with and without TiO_2_ electron selective contact layer and InP passivation layer highlighting the impact of different layers on the InGaAsP photovoltaic performance. The inset in the dark *J–V* curve shows the *J*_01_ component of the dark current obtained by fitting the dark current using a double diode model.

The recombination of photogenerated charge carriers at the surface and bulk of the solar absorber material represents a significant limitation in the efficiency of solar cells. Mitigating such recombination can be achieved through an individual or combined effects involving the reduction of trap states due to dangling bonds present at the surface through chemical passivation and/or the reduction of photogenerated charge carriers available for recombination through field‐effect passivation [55]. The remarkable PV performance of the TiO_2_/InP/InGaAsP solar cell results from the combined effect of these processes facilitated by both the InP and TiO_2_ ESC layer. To elucidate the individual contributions of these layers, devices were fabricated with only TiO_2_, only InP, and with both layers, and their PV performances were evaluated (Figure 3 and Table 3). The light *J–V* curves (Figure 3a) reveal that individually, these layers on InGaAsP are not sufficient to achieve such excellent PV performance.

**TABLE 3:** Summary of the performance parameters of various InGaAsP solar cells to compare the role of TiO_2_ and InP layers.

Device	***V***_**oc**_ **(mV)**	***J***_**sc**_ **(mA cm** ^**−2**^ **)**	*FF* (%)	*η* (%)
ITO/InGaAsP	522	30.1	68.8	10.8
ITO/TiO_2_/InGaAsP	584	38.6	71.7	16.5
ITO/InP/InGaAsP	596	36.4	70.8	15.3
ITO/TiO_2_/InP/InGaAsP	657	40.4	71.8	19.0

In the solar cell with the optimal InP layer (ITO/InP/InGaAsP), the *V*_oc_ and *J*_sc_ improve significantly from 522 to 596 mV and from 30.1 to 36.4 mA cm^−2^, respectively, compared to the ITO/InGaAsP reference cell. Similarly, in the solar cell with only the TiO_2_ ESC layer (ITO/TiO_2_/InGaAsP), the *V*_oc_ and *J*_sc_ improve significantly from 522 to 584 mV and from 30.1 to 38.6 mA cm^−2^, respectively, compared to the ITO/InGaAsP reference cell. Interestingly, the cell with only the TiO_2_ ESC layer has a significantly higher *J*_sc_ value compared to the cell with only the InP layer. The higher *J*_sc_ in the ITO/TiO_2_/InGaAsP cell compared to the ITO/InP/InGaAsP cell originates from the selective transport of photogenerated electrons and simultaneous blocking of holes facilitated by the TiO_2_ ESC layer. This significantly improves the photogenerated electron collection and transport from the InGaAsP layer, resulting in a higher *J*_sc_. On the other hand, the band alignment of InP with InGaAsP is not favorable for carrier‐selective characteristics (as will be detailed in the band energetics section), leading to a lower *J*_sc_. This observation is further corroborated by EQE measurements (Figure 3b), where cells with only the TiO_2_ ESC layer have substantial enhancement in overall carrier collection and transport compared to cells with only the InP layer. Table S4 presents a comparison of the *J*_sc_ values obtained from the light *J–V* measurements and those calculated by integrating the EQE. The differences between these values remain within an acceptable margin of less than 5%, which is typical for PV device characterization. These minor discrepancies are primarily attributed to spectral mismatches between the AM1.5G reference spectrum used in EQE measurements and the output spectrum of the solar simulator, particularly in the near‐infrared region.

It is noteworthy that the *V*_oc_ of ITO/InP/InGaAsP and ITO/TiO_2_/InGaAsP cells have significantly higher values compared to the reference (ITO/InGaAsP) cell. This improvement is due to enhanced surface passivation enabled by both layers, as reflected by a one‐order‐of‐magnitude reduction in the *J*_01_ component of dark current in both cases (Figure 3c). However, the mechanism of surface passivation or reduction in *J*_01_ differs in both cases. In the case of the InP layer, surface passivation is enabled by the chemical passivation of the surface dangling bonds at the InGaAsP surface. Meanwhile, the TiO_2_ ESC layer passivates the InGaAsP surface through a field effect passivation mechanism, reducing the probability of photogenerated carrier recombination at the interface by selectively transporting electrons and simultaneously blocking holes [56]. Interestingly, the chemical passivation of the InGaAsP surface enabled by InP layer could be further complemented by the field‐effect passivation provided by the TiO_2_ ESC layer, as evident from the additional one‐order‐of‐magnitude reduction in the recombination current (Figure 3c). This synergistic combination of the InP and TiO_2_ ESC layers resulted in a significant enhancement in *V*_oc_ and *J*_sc_, increasing from 522 mV and 30.1 mA cm^−2^ in the reference cell to 657 mV and 40.4 mA cm^−2^ in the cell with the optimal InP and TiO_2_ layers, respectively. Such combinations of passivating and carrier‐selective layers have been demonstrated previously to be effective in achieving high solar cell efficiencies [57, 58, 59].

### 2.3 Impact of InP Passivation from Theoretical Modeling

**FIGURE 4:**
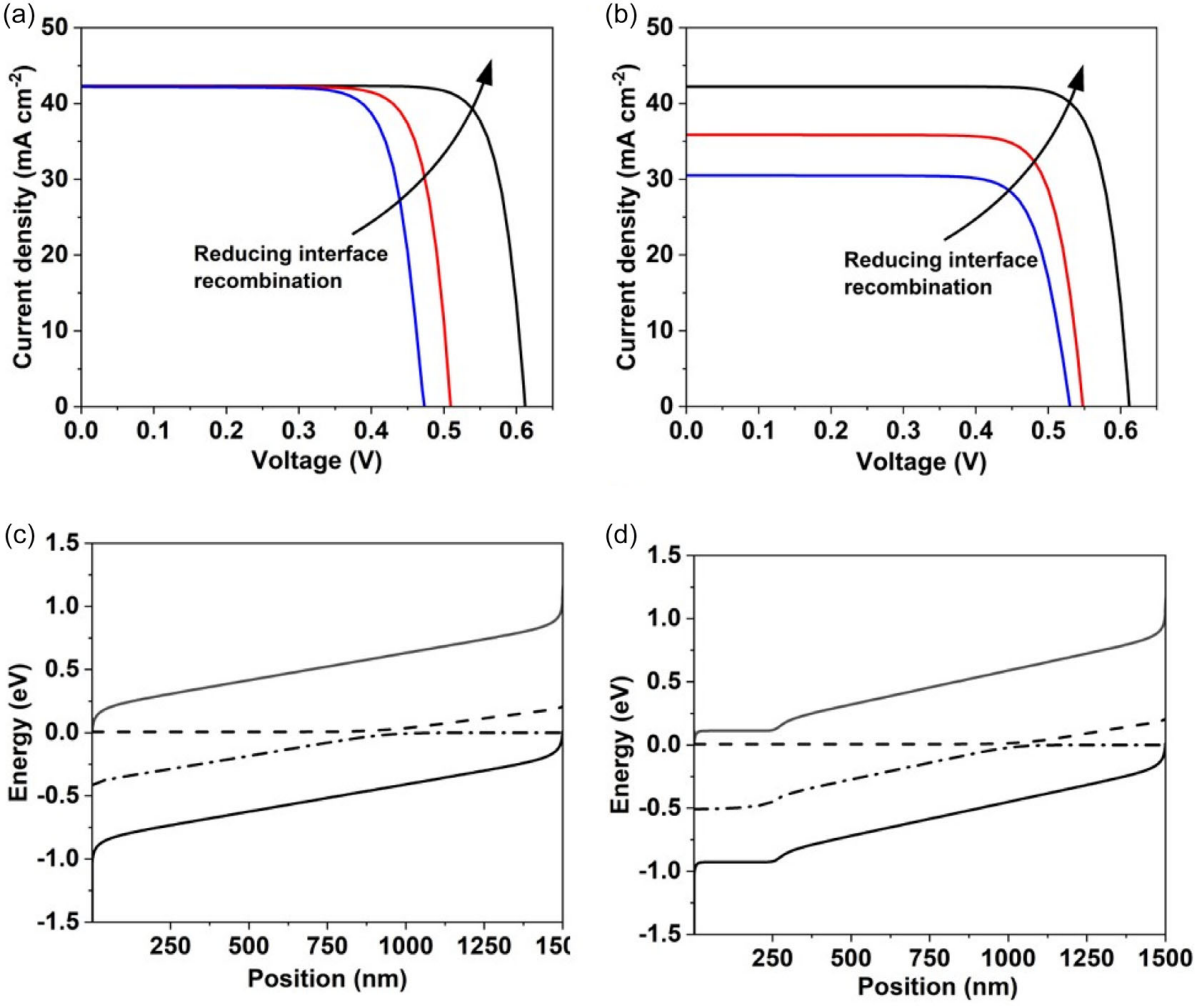
Simulated influence of interface recombination on *J–V* curves of InGaAsP solar cell and the corresponding energy bands in the InGaAsP absorber layer. In (a) the InGaAsP layer is intrinsic, and we observe no significant influence of interface recombination on short circuit current. In (b) the same device with an n^+^ diffused region in the InGaAsP absorber at the TiO_2_ interface. In the presence of the dopant space charge region, we observe a loss in short circuit current in addition to open‐circuit voltage. (c) In a completely intrinsic InGaAsP layer, there is no space charge to screen the built‐in electric field, which therefore contributes to charge collection via electron/hole drift. (d) In a device with a near‐surface n^+^ diffused region at the sunward‐facing TiO_2_ electron contact, the dopant space charge screens the built‐in field.

The introduction of the InP passivation layer on the InGaAsP results in an increase in both *V*_oc_ and *J*_sc_, as evident in Figure 2c. The enhancement in *V*_oc_ is likely attributed to the reduction in surface defect density (*D*_*it*_) at the InGaAsP interface facilitated by the presence of the InP interface passivation layer [41]. However, the improvement in *J*_sc_ due to interface passivation is a relatively uncommon observation in conventional PV technologies [60, 61, 62, 63]. To explain this observation, we carried out numerical simulations of our device, which reveal a relatively complex picture of charge collection in the InGaAsP layer. Foremost, the experimentally observed change in *J*_sc_ in response to InP passivation is inconsistent with simulated *J*_sc_ with an entirely intrinsic (undoped) InGaAsP layer. As evident in simulated light *J–V* curve of the device (Figure 4a), there is improvement in the device *V*_oc_ in response to changing surface *D*_*it,*_ but there is no significant change in the device *J*_sc_. In an intrinsic InGaAsP layer, there would be insufficient space charge to screen the work–function difference between the p^+^ InP (at rear) and the Ag/ITO electrodes (at front). Consequently, a strong electric field (∼10^5^ V m^−1^) across the entire intrinsic InGaAsP layer would be established by the work–function difference between the electrodes (Figure 4c). This electric field would result in a strong drift contribution to electron and hole collection. Even a high level of interface recombination at the intrinsic InGaAsP surface would not be sufficient to reduce short‐circuit charge collection, as shown in the simulated *J–V* curves of Figure 4a. Hence, for an intrinsic InGaAsP absorber, there would be *V*_oc_ losses in response to interface recombination, but negligible changes in *J*_sc_.

Consequently, for *J*_sc_ to be reduced by increasing interface recombination, there must be space charge region in the InGaAsP layer to screen this electric field. If we incorporate an n^+^ dopant region at the InGaAsP interface into our model (Figure S6), we find that the dopant profile screens the electric field in the near‐surface region (Figure 4d). Thus, in the presence of high interface recombination, photogenerated carrier collection by the contacts is significantly reduced owing to preferential recombination within the doped region at the interface. Consequently, *J*_sc_ is impacted significantly by the changes in the interface recombination in the presence of an n^+^ doped interface region in InGaAsP layer (Figure 4c). To further elucidate this effect, Figure S7 plots carrier collection probability as a function of position in a device with a 250 nm‐thick n^+^ doped region. We observe a significant reduction in charge collection efficiency with the doped region near the InGaAsP interface, which does not occur without surface doping or with low interface recombination.

For a possible source of formation of space charge region, we speculate that this could be due to sputtering‐induced damage caused during ITO sputtering. It was previously observed that ITO sputtering forms a buried junction on the III–V semiconductor surface due to plasma‐induced damage, creating a charge inversion (n‐type) region due to formation of donor‐like defects [42, 64, 65]. The ITO sputtering could induce donor‐like defects on the surface of InGaAsP and thus create an n‐type region on the surface. It is interesting to note that the charge inversion region created due to sputtering induced damage extends only couple of hundred nanometers into the semiconductor surface which is in line with our current assumption of dopant profile in Figure S6 [64, 66]. However, further studies are needed to get deeper insights into the mechanism.

### 2.4 Band Energetics at the Interface

**FIGURE 5:**
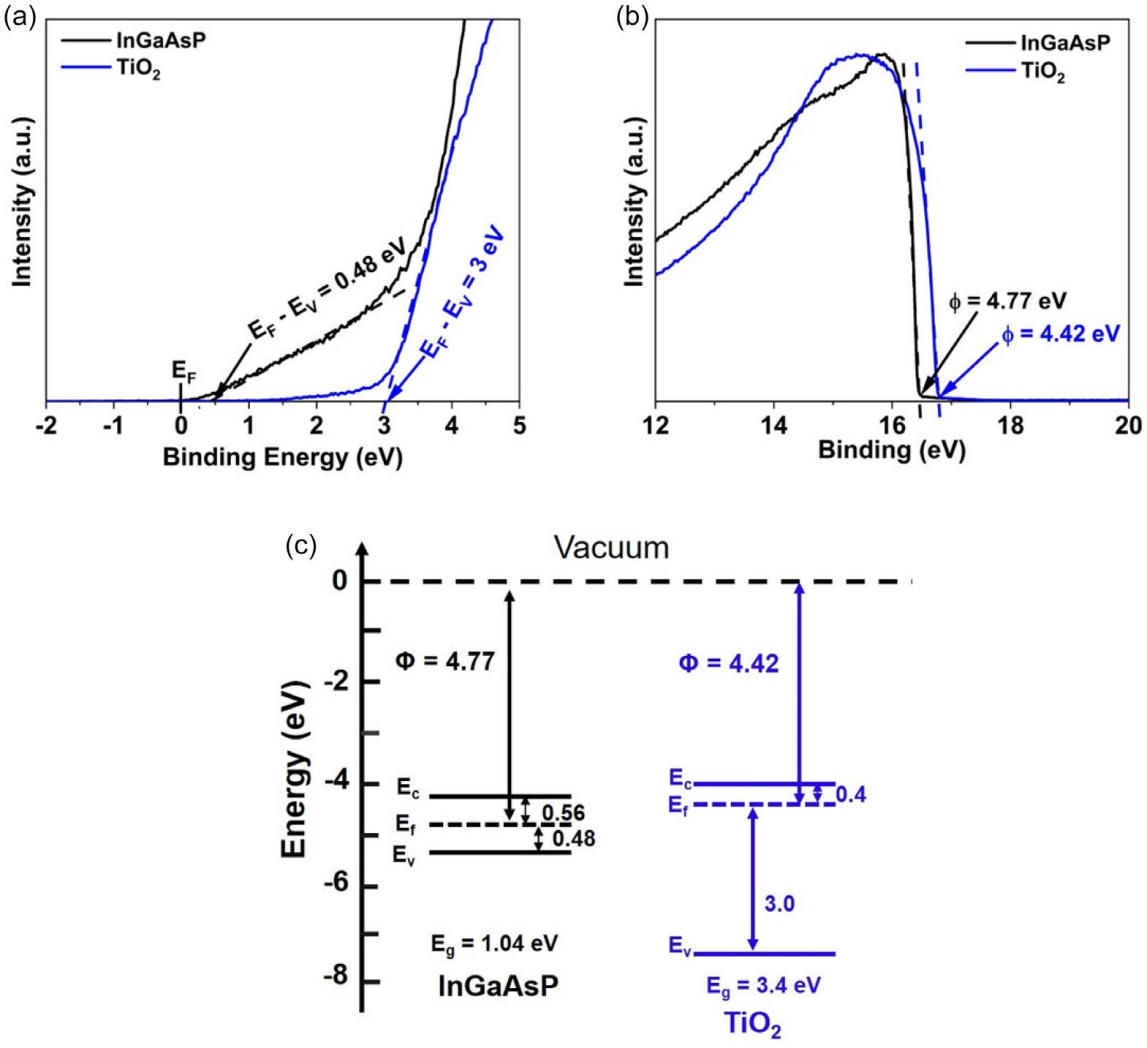
X‐ray and ultraviolet photoelectron spectroscopy of InGaAsP and TiO_2_. (a) Secondary electron cutoff spectrum and (b) valence band spectrum of InGaAsP and TiO_2_. (c) Schematic of the band diagram at the InGaAsP/TiO_2_ interface derived from X‐ray and ultraviolet photoelectron measurements.

To gain an in‐depth understanding of the electron‐selective characteristics of TiO_2_, XPS and UPS measurements were conducted on TiO_2_, InP, and InGaAsP, as illustrated in Figure 5 and Figure S8. In Figure 5a, the XPS valence band spectrum of InGaAsP and TiO_2_ is presented. The determination of the position of the valence band maximum (*E*_*V*_) in relation to the Fermi level (*E*_*F*_) involved extrapolating the linear portion of the valence band spectrum to the horizontal axis. The *E*_*V*_ values for InGaAsP and TiO_2_ were identified at 0.48 and 3 eV relative to *E*_*F*_, respectively. Moreover, by leveraging the bandgap information of a material, the relative position of the conduction band minima (*E*_*C*_) could be ascertained using the equation *E*_*g*_ = *E*_*C*_ – *E*_*V*_. Considering bandgaps of 1.04 and 3.4 eV for InGaAsP and TiO_2_ (Figure S9), respectively, the *E*_*C*_ was determined to be 0.56 and 0.4 eV above the *E*_*F*_ for InGaAsP and TiO_2_, respectively. Likewise, the work function of these materials could be ascertained from the UPS spectra using the provided equation.

(3) $$Work\;\text{function}\;\left(\Phi \right)=hv-(E_{cut-off}-\;E_{F})$$

Here, *hν* represents the energy of the He–I source (21.2 eV), *E*_*cut‐off*_ signifies the cutoff of the tail at the higher‐binding energy end of the UPS spectrum, and *E*_*F*_ denotes the Fermi energy, positioned at 0 eV.

Utilizing the information from the UPS spectra (Figure 5b) and Equation (3), the derived work function for InGaAsP is 4.77 eV. This implies that the *E*_*F*_ of InGaAsP is positioned 4.77 eV below the vacuum level. Similarly, the calculated work function for TiO_2_ is 4.42 eV.

Based on the acquired data, the band diagram of InGaAsP and TiO_2_ is constructed and depicted in Figure 5c. The conduction band offset Δ*E*_*C*_ = [(*E*_*C*_)_*InGaAsP*_ − (*E*_*C*_)_*TiO2*_] is only 0.19 eV, while the valence band offset Δ*E*_*V*_ = [(*E*_*V*_)_*InGaAsP*_ − (*E*_*V*_)_*TiO2*_] is 2.17 eV. The small conduction band offset promotes the efficient transfer of photo‐generated electrons from the InGaAsP absorber layer to the TiO_2_ ESC layer. Importantly, Δ*E*_*C*_ is larger than the thermal energy of electrons at room temperature, preventing electron transfer back into the InP layer. Additionally, the substantial valence band offset of 2.17 eV at the InGaAsP/TiO_2_ interface hinders the transfer of photo‐generated holes to the TiO_2_ ESC layer. This highlights that TiO_2_ functions as an effective electron‐selective and hole‐blocking layer for photo‐generated carriers in InGaAsP. On the other hand, InP offers very low conduction band (0.26 eV) while simultaneously offering low valence band offset (0.56 eV) resulting in its limited capability in blocking holes (Figure S8).

### 2.5 Semi‐Transparent Perovskite Solar Cell Characterization

**FIGURE 6:**
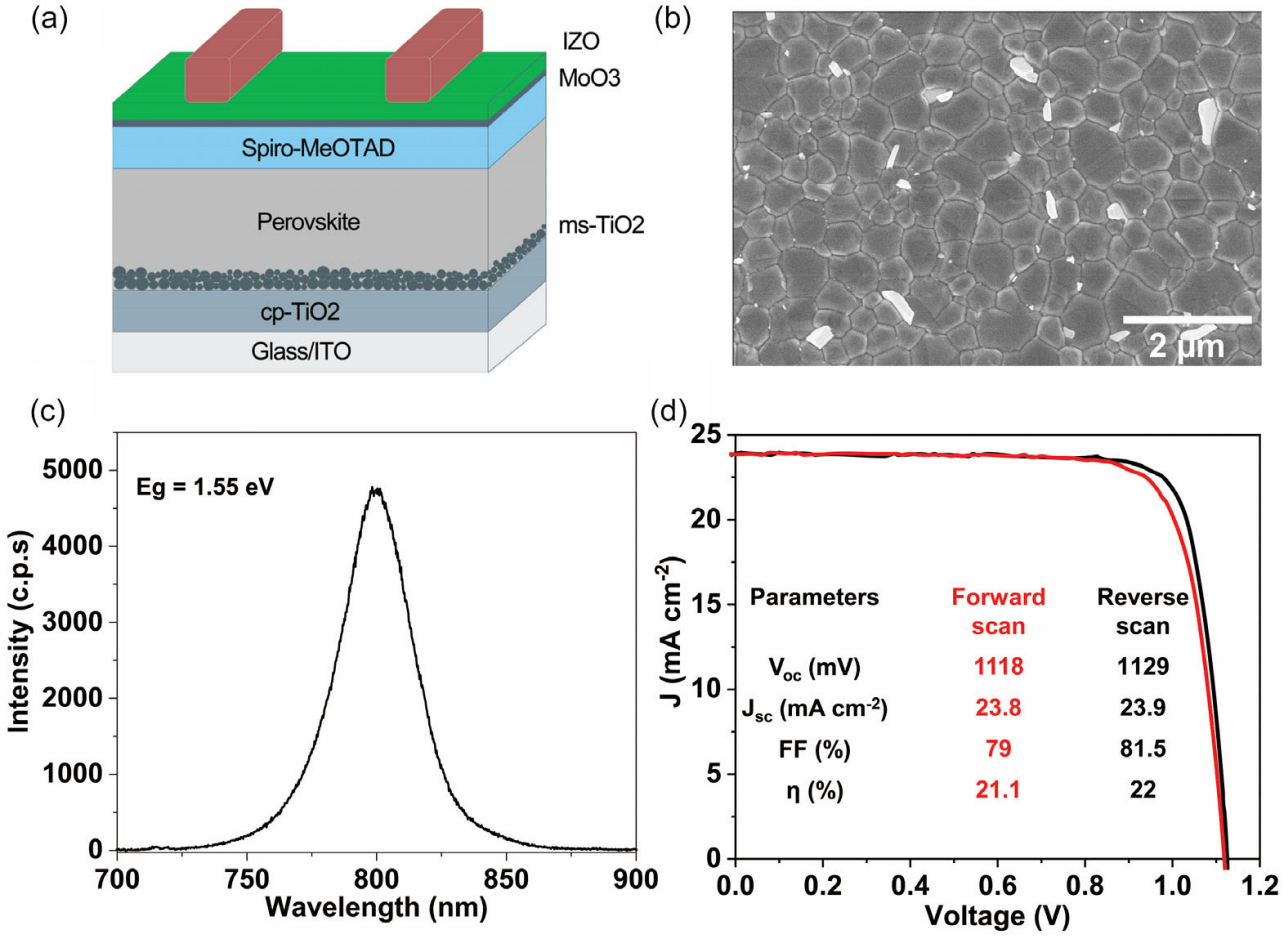
Characterization of semi‐transparent perovskite solar cell. (a) Schematic illustration of semitransparent perovskite solar cell depicting different materials in the structure. (b) Scanning electron microscopy image and (c) photoluminescence spectra of the perovskite layer. (d) Light *J–V* curve of the semi‐transparent perovskite solar cell, with an inset depicting key photovoltaic performance metrics.

A semi‐transparent perovskite solar cell, compatible with the existing InGaAsP bottom cell in a tandem configuration, was fabricated, and its characteristics are displayed in Figure 6. The schematic representation of the semitransparent perovskite solar cell with different constituent layers is presented in Figure 6a. This cell comprises compact TiO_2_ and mesoporous TiO_2_ as an electron transport layer, a Spiro‐MeOTAD hole transport layer and MoOx is buffer layer to prevent the sputter damage to the hole transport layer [67]. The perovskite solar absorber material, free from methylammonium, has the composition FA_0.9_Cs_0.1_PbI_3_. The surface morphology of the perovskite film was investigated using SEM, and its corresponding micrograph is depicted in Figure 6b. The micrograph reveals perovskite crystals with an average grain size of around 850 nm. The larger crystal size of perovskite materials is advantageous in reducing grain boundaries, thereby aiding in the suppression of non‐radiative recombination and carrier scattering [68]. Moreover, the grain boundaries appear smooth, indicating the absence of segregation of other phases. The bright features observed on the surface of the perovskite films correspond to 4‐methylphenethylammonium chloride (4‐MPEACl). The optical properties of the perovskite films are assessed through PL and ultraviolet–visible (UV–vis) absorption spectra. Figure 6c presents the PL spectra of the perovskite film, showing very high PL emission indicative of superior radiative recombination in the films, which would be advantageous for achieving a high *V*_oc_. The PL emission is centered around 797 nm, corresponding to the optical bandgap of 1.55 eV. This bandgap of the perovskite film is suitable for the bottom InGaAsP cell with a 1.04 eV bandgap [69]. The UV–vis spectra of the semitransparent perovskite cells in Figure S10 reveal an onset of absorption around 780 nm, aligning with the PL emission at a similar wavelength. Furthermore, solar cells exhibit excellent transparency of up to 80% in the infrared, highlighting their applicability as the top cell in a tandem configuration. The PV performance of the semitransparent perovskite solar cell in a normal n–i–p structure is evaluated and presented in Figure 6d. The champion cell demonstrated an efficiency of 22.0% (reverse scan)/21.1% (forward scan) and a steady‐state performance of 21.7% and showed no signs of efficiency degradation for more than 350 h (Figure S11). Notably, the solar cell exhibited a commendable *J*_sc_ of 23.9 mA cm^−2^, considering its semitransparent nature. Additionally, the solar cells showed very low hysteresis in forward and reverse scans, demonstrating superior material quality.

### 2.6 InGaAsP/Perovskite Mechanical Tandem Solar Cell Characterization

**FIGURE 7:**
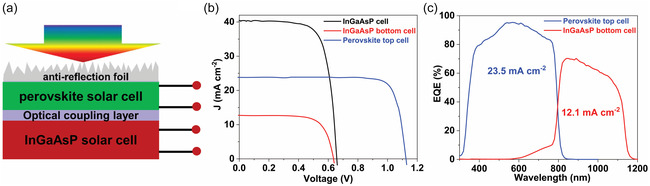
Characterization of the perovskite/InGaAsP tandem solar cell. (a) Schematic of the perovskite/InGaAsP solar cell. (b) Light *J–V* characteristics and (c) external quantum efficiency of perovskite/InGaAsP tandem solar cell.

We employed a low‐to‐moderate bandgap perovskite composition in 4‐terminal perovskite/InGaAsP tandem solar cells. In the absence of the current matching requirement in the 4‐terminal tandem configuration, this perovskite top cell with a low‐to‐moderate bandgap can achieve efficiency close to the optimal level while potentially providing significantly enhanced operational stability compared to high bandgap perovskites with mixed‐halide compositions [70]. Figure 7a depicts a schematic of the 4‐terminal mechanically stacked perovskite/InGaAsP tandem solar cells. To reduce reflectance losses at the front surface and between the two cells, we applied an anti‐eflection foil and optical coupling liquid, respectively. The light *J–V* curve for each subcell is displayed in Figure 6b, and the PV performance is summarized in Table 4. The champion semi‐transparent perovskite top cell demonstrated an efficiency of 22.0%. When subjected to a semi‐transparent perovskite cell filter, which maintained all layers identical to the active semi‐transparent perovskite cells, the bottom InGaAsP cell, which originally had an efficiency of 19.0%, has an efficiency of 5.7%. Figure 7c displays the EQE spectrum of both the semi‐transparent perovskite cell and the InGaAsP cell under the perovskite filter. The EQE results exhibit remarkable consistency with the light *J–V* results of the devices. The integrated *J*_sc_ for the semi‐transparent perovskite top cell reaches 23.6 mA cm^−2^, while the integrated *J*_sc_ for the InGaAsP cell under the semi‐transparent perovskite is 12.1 mA cm^−2^. The overall efficiency of the 4‐terminal perovskite/InGaAsP tandem solar cell is determined to be 27.7%, accompanied by an exceptional *V*_oc_ of 1.7 V. Notably, this efficiency is comparable to the highest reported values for experimental tandem devices incorporating perovskites [71, 72] and the highest reported for perovskite/III–V tandems to our knowledge [16].

**TABLE 4:** Photovoltaic performance of InGaAsP/perovskite tandem solar cell.

Device	***V***_**oc**_ **(mV)**	***J***_**sc**_ **(mA cm** ^**−2**^ **)**	*FF* (%)	*η* (%)
InGaAsP cell	657	40.3	71.8	19.0
Semitransparent perovskite cell	1125	23.9	81.5	22.0
InGaAsP cell under perovskite cell	634	12.7	70.4	5.7
Tandem efficiency				27.7

### 2.7 Photovoltaic Power and Fill Factor Loss Analysis at the Maximum Power Point

**FIGURE 8:**
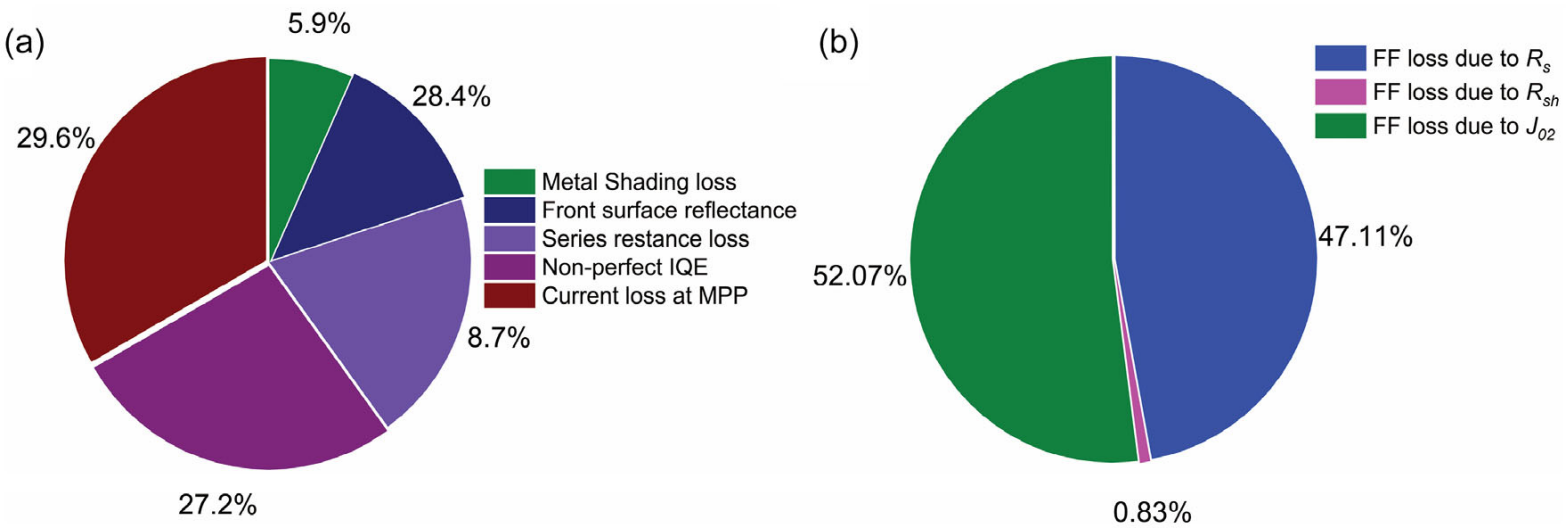
Pie‐chart representation of the percentage of total (a) power losses and (b) fill factor losses due to different mechanisms.

To identify potential areas for improvement in the performance of our tandem cell, we conducted an extensive analysis of losses of InGaAsP cell at the maximum power point (MPP), drawing upon the methodology established by Aberle et al. [73]. While this loss analysis excludes the consideration of *V*_oc_ loss and focuses solely on the calculation of current and power loss at the MPP, it provides with measurable values for various other key loss mechanisms. These include power losses attributed to series resistance and shunt resistance, optical losses arising from front metal shading and front surface reflectance, as well as recombination losses at the MPP due to nonperfect IQE and forward‐bias current loss. Insights into these losses serve as a guide for future experiments with the goal of enhancing the efficiency of the presented solar cell in future studies. Figure 8a and Table S5 outline the various power loss mechanisms that impede the efficiency of our ITO/TiO_2_/InP/InGaAsP solar cell. Clearly, recombination losses (4.8 mW cm^−2^) and optical losses (2.9 mW cm^−2^) are the primary contributors to power losses. The substantial recombination loss implies that enhancing the passivation quality at both interfaces (front and rear) is imperative for achieving improved efficiency.

In the current device, the rear side, with only a highly doped AuZn rear ohmic contact, is anticipated to be a significant contributor to carrier recombination. Suppression of recombination could likely be achieved by introducing a hole‐selective passivating contact layer. This not only would diminish recombination at the rear side of the device but also enhance carrier collection at the rear side. As indicated by the EQE data in Figure S5, a significant portion of the current losses in the device is attributed to poor collection efficiency at higher wavelengths. Moreover, the reduction of optical losses could be achieved through the optimization of metal finger grids to minimize shading losses and the incorporation of an antireflective coating to mitigate front surface reflectance.

Similarly, it is crucial to quantify the *FF* losses in the cell, providing valuable insights for further enhancing the efficiency of the device. The different *FF* losses are obtained from the equations below and shown in Figure 8b and Table S5 [74].

(4) $$FF_{0}=\;FF+\;\frac{J_{mpp}^{2}\;R_{s}}{V_{oc}J_{sc}}+\;\frac{{\left(V_{mpp}+J_{mpp}R_{s}\right)}^{2}}{V_{oc}J_{sc}R_{sh}}$$

(5) $$\Delta FF_{R_{s}}=\;\frac{J_{mpp}^{2}\;R_{s}}{V_{oc}J_{sc}}$$

(6) $$\Delta FF_{R_{sh\;}}=\;\frac{{\left(V_{mpp}+J_{mpp}R_{s}\right)}^{2}}{V_{oc}J_{sc}R_{sh}}$$

The terms in the equation represent various parameters and losses in the solar cell, where *J*_sc_ and *J*_*mpp*_ denote the short‐circuit current density and current density at the MPP, respectively. *V*_oc_ and *V*_*mpp*_ represent the open‐circuit voltage and voltage at the MPP, respectively. *R*_*s*_ and *R*_*sh*_ are the series and shunt resistances of the cell obtained by fitting the dark current using a two‐diode model. *FF*_0_ represents the FF without any resistances in the cell, while Δ*FF*_*Rs*_ and Δ*FF*_*Rsh*_ denote the fill factor loss due to series and shunt resistances, respectively.

Beyond resistances, the FF losses in the cell are also influenced by recombination in the depletion region (*J*_02_). To quantify the fill factor loss caused by depletion region recombination (*FF*_J02_), we initially computed the upper limit of fill factor (*FF*_J01_) using an analytical equation proposed by Green et al. [75]. The *FF* loss due to recombination in the depletion region is simply the difference between $FF_{J_{01}}$ and *FF*_0_ *.*

(7) $$FF_{J_{01}\;}=\;\frac{v_{oc}-\ln\;(v_{oc}+0.72)}{v_{oc}+1}$$

where

(8) $$v_{oc}=\;q\frac{V_{oc}}{nkT}$$

and *q* is the elementary electronic charge, *n* is the diode ideality factor, *k* is the Boltzmann constant, and *T* is the temperature at which measurements were performed (298 K).

The ITO/TiO_2_/InP/InGaAsP cell has a maximum achievable FF of 83.9%, while the achieved *FF* is 71.8%, resulting in a FF loss of 12.1% (Table S5). Within this, series and shunt resistance losses contribute 5.7% and 0.1%, respectively, and the remaining 6.3% is attributed to *J*_02_ recombination loss. Notably, the majority of the fill factor loss is associated with series resistance and *J*_02_ recombination, indicating the importance of addressing depletion region recombination current and contact resistances. By effectively mitigating these losses, the InGaAsP solar cell holds the potential to exceed 27% efficiency. Such improvements would also enhance the efficiency of the tandem solar cell, extending beyond 30%. The proposed tandem solar cell design holds the promise of achieving impressive efficiency levels, thereby opening doors for high‐efficiency thin‐film flexible tandem solar cells, a feat difficult to achieve in Si/perovskite tandem solar cell technology.

## 3 Conclusion

In conclusion, we have developed a low‐bandgap InGaAsP heterojunction solar cell achieving an efficiency of 19% and an open circuit voltage of 657 mV, with performance comparable to the best reported InGaAsP devices. In contrast to prior studies that have largely focused on homojunction design, epitaxy and postprocessing optimization, this work introduces a heterojunction architecture that leverages interface engineering to enhance performance. A significant innovation in this study is the synergistic integration of a thin InP passivation layer with a TiO_2_ electron‐selective contact, which enables effective surface passivation while supporting efficient carrier extraction. While elements of this approach have been previously explored individually, we have combined them in this work to enhance device performance. Moreover, we have demonstrated a mechanically stacked, four‐terminal tandem solar cell that pairs the low‐bandgap InGaAsP bottom cell with a semi‐transparent perovskite top cell, delivering a remarkable efficiency of 27.7% and open circuit voltage of 1.7 V. To the best of our knowledge, this represents one of the rare demonstrations of a perovskite/III–V tandem cell, highlighting the potential of such combination for hybrid multijunction PVs. Through detailed loss analysis, we have identified the dominant losses limiting device performance, revealing clear pathways to exceed 19% efficiency in the InGaAsP heterojunction cell and 30% in tandem configurations. This quantitative insight into loss mechanisms provides design guidance beyond prior reports. These findings, with the bandgap tunability of lattice‐matched InGaAsP, establish a versatile platform for bandgap‐engineered perovskite/III–V tandem design and opens new avenues for the development of III–V multijunction solar cells through nonepitaxial growth techniques.

## Conflicts of Interest

The authors declare no conflicts of interest.

## Supporting information

Supplementary Material

## Data Availability

Data available from corresponding author upon request.
