# Supplementary material for: Achieving 27.7% Efficiency with a Mechanically Stacked, Four‐Terminal Perovskite/InGaAsP Tandem Solar Cell
Source: Small Sci. 2026 May 9;6(5):e70298. doi: 10.1002/smsc.70298 (PMC13156521; doi:10.1002/smsc.70298)
Supplement: Supplementary file 1 — Supplementary Material [file SMSC-6-e70298-s001.pdf]

Supporting Information

Achieving 27.7% Efficiency with a Mechanically Stacked, Four-Terminal Perovskite/InGaAsP Tandem Solar Cell

Bikesh Gupta[1], The Duong[2], Tuomas Haggren[1,3], Daniel Walter[2], Julie Tournet[2*], Azul Osorio Mayon[2], Chennupati Jagadish[1,3], Hark Hoe Tan[1,3], Siva Karuturi[2*]

[1]Department of Electronic Materials Engineering, Research School of Physics, The Australian National University, Canberra, ACT 2600, Australia.

[2]School of Engineering, The Australian National University, Canberra, ACT 2600, Australia.

[3]ARC Centre of Excellence for Transformative Meta-Optical Systems, Research School of Physics, The Australian National University, Canberra, ACT 2600, Australia.

*Corresponding email: julie.tournet@anu.edu.au, siva.karuturi@anu.edu.au

Experimental Section

Materials

Formamidinium iodide, 4M-PEACl, and n-octylammonium bromide were obtained from Great Cell solar materials. Lead iodide was sourced from TCI Co., LTD. Lum-Tech provided Spiro-MeOTAD. The refractive index matching layer (series AA, refractive index = 1.414) was acquired from SPI Supplies. Alfa Aesar supplied tin (IV) oxide, 15% in H_2O colloidal dispersion. Other materials were purchased from Sigma-Aldrich. All chemicals were utilized without additional purification.

InGaAsP growth

The InGaAsP photoabsorber layer was grown using metal-organic chemical vapor deposition in an AIXTRON horizontal flow reactor. Trimethylindium, trimethylgallium, arsine, and phosphine, were utilized as the precursors for the In, Ga, As, and P, respectively. This InGaAsP layer was deposited on p-doped (Zn: 2.4–3.5 × 10^{18} cm^{-3}) InP (100) substrates. Following an initial bake-out at 700 °C to eliminate the native oxide, a 200 nm InP buffer layer was grown at 650 °C and a reactor pressure of 180 mbar. The buffer was p-doped, with a hole concentration of 9.9 × 10^{17} cm^{-3}. Subsequently, an undoped 1.5 μm-thick InGaAsP

layer was grown at 650 °C as a random alloy, with all gases fed simultaneously. The V/III ratio was maintained at a high value of 591. The target composition of the quaternary material was $In_{0.70}Ga_{0.30}As_{0.65}P_{0.35}$, designed to achieve a bandgap of 1 eV while being closely lattice-matched to InP. Likewise, various thicknesses of undoped InP passivating layers were grown in the same reactor at 650 °C, using only trimethylindium and phosphine.

InGaAsP heterojunction solar cell fabrication

First, ohmic contacts on the rear side of InGaAsP wafers were produced through the sputtering process (ATC 2400, AJA International Inc.) using 20 nm Zn and 100 nm Au films. Subsequently, the samples underwent annealing at 400 °C for a duration of 40 minutes in a constant flow of forming gas composed of 5% H_2 and 95% N_2. Next, the TiO_2 electron-selective contact (ESC) layer was deposited through the atomic layer deposition (ALD) process using a Veeco Savannah 100 system. $Ti[OCH(CH_3)_2]_4$ (titanium isopropoxide) and H_2O served as the titanium and oxygen precursors, respectively. Different thicknesses of TiO_2 were achieved by adjusting the number of deposition cycles, maintaining a deposition rate of 0.016 nm per cycle. Before TiO_2 depositions, a 5% hydrofluoric acid cleaning step for 45 seconds was performed on all samples to eliminate any native oxides present on the surface.

Subsequently, a transparent conducting oxide (ITO: In_2O_3:SnO = 90%:10%) was sputtered onto the TiO_2 layer at room temperature using an ATC 2400 system from AJA International Inc. The sputtering was carried out in an argon atmosphere (20 sccm, 1.5 mTorr) with an RF input power of 60 W. The thickness of the ITO layer was adjusted to maintain a total thickness of 70 nm for both TiO_2 and ITO, ensuring minimal reflectance around 600 nm. Finally, silver finger grids with a width and pitch of 11 and 490 μm, respectively, were created through a process involving photolithography, e-beam evaporation, and a lift-off technique. After fabrication, the device edges were cleaved to define an active area of 5 mm × 5 mm. In a similar manner, InGaAsP solar cells featuring passivating InP layers were fabricated using the same process, with or without the TiO_2 ESC layer.

Semi-transparent perovskite solar cell fabrication

Semi-transparent perovskite solar cells were fabricated following the protocol as described in Ref [1]. In summary, perovskite cells were manufactured on indium-doped tin oxide (ITO) glass substrates. Prior to cell fabrication, the ITO glass substrates underwent a multistep

cleaning procedure involving sequential sonication in acetone, isopropyl alcohol (IPA), and ethanol, each lasting 15 minutes. Subsequently, they underwent an additional cleaning step through exposure to UV ozone for 15 minutes. The substrates were then promptly transferred to a glove box purged with N_2 for cell fabrication. Initially, a SnO_x electron transport layer was deposited onto an ITO glass substrate using a diluted colloidal dispersion of SnO_2 in deionized (DI) water (mixed at a 1:3 volume ratio). The colloidal precursor of SnO_x was spin-coated onto the substrates at 3000 rpm for 30 seconds and then subjected to annealing at 150 °C for a duration of 30 minutes. The precursor for the perovskite was prepared by combining 1.3 M PbI_2, 1.3 M formamidinium iodide (FAI), and 0.13 M CsCl in 1 mL of dimethylformamide (DMF)/dimethyl sulfoxide (DMSO) (with a volume ratio of 4:1). Within the perovskite precursor solution, 2% of 4 M phenethylammonium chloride (PEACl) was mixed. The deposition of perovskite onto the substrates was performed by spinning a 40 µL solution at 1000 rpm for 10 seconds (with an acceleration rate of 200 rpm s^{-1}) and then at 4000 rpm (with an acceleration rate of 1000 rpm s^{-1}) for 25 seconds. Five seconds before the program concluded, chlorobenzene (120 µL) was promptly deposited onto the centre of the substrates. Subsequently, an annealing process was conducted on a hotplate for 10 minutes at 150 °C and an additional 10 minutes at 100 °C. A solution of n-octylammonium iodide (2 mg mL^{-1} in 2-propanol) was spin-coated onto the substrates at 5000 rpm for 30 seconds. Following this, the substrates underwent additional annealing at 100 °C for a duration of 5 minutes. The deposition of the Spiro-MeOTAD layer onto the perovskite films was carried out by spinning a solution of Spiro-MeOTAD (40 µL, concentration: 72 mg mL^{-1}) in chlorobenzene, along with Li-TFSI (17.5 µL, concentration: 520 mg mL^{-1} in acetonitrile) and 4-tBp (28.5 µL), at 3500 rpm for 30 seconds. Following this, a thermal evaporation process was employed to deposit a 10 nm layer of MoO_3 onto the substrate at a pressure of $\approx 5 \times 10^{-7}$ Torr. Subsequently, a 30 nm layer of indium zinc oxide (IZO) was sputtered onto the substrates via radio frequency sputtering, utilizing a shadow mask with an active area of 0.36 cm^2. Finally, Au metal grids were deposited on top of the IZO layers through thermal evaporation.

Solar cell characterizations

The light *J-V* characteristics of the semi-transparent perovskite solar cells were evaluated using a solar simulator, model #WAVELABS SINUS-220, coupled with a potentiostat source, Autolab PGSTAT302N. The light intensity was standardized to one sun (100 mW cm^{-2}, AM1.5G) through calibration utilizing a certified Fraunhofer CalLab reference cell.

Throughout the measurements, an opaque mask with a 1 cm^2 aperture was employed. The photovoltaic performance of the InGaAsP solar cells was measured under simulated one sun illumination (100 mW cm^{-2}) using a Newport solar simulator equipped with an AM1.5G filter. A commercial Si photodiode was employed to calibrate the illumination intensity of the solar simulator. Dark current-voltage measurements were conducted using an Agilent N1294A source measuring unit equipped with an ultra-low noise filter. The external quantum efficiency of the solar cells was measured using a NewSpec IQE-200 AC system, which was calibrated before each measurement. All solar cell measurements were carried out at 25 °C under ambient conditions. For the measurement of a large-area perovskite solar cell and an InGaAsP solar cell in a four-terminal tandem configuration, the 0.5 cm^2 InGaAsP solar cell was positioned directly beneath the semi-transparent perovskite top cell. A refractive index matching liquid was introduced between the two cells, and a textured foil was placed on top of the perovskite cell.

Material characterizations

High-resolution transmission electron microscopy (HRTEM) analysis was conducted using a JEOL 2100F instrument operated at 200 keV for cross-sectional characterization of the InGaAsP solar cell. A focused ion beam (FEI Helios 600 NanoLab) equipped with a Ga ion beam was employed to prepare the cross-sectional HRTEM lamella. Before milling, a layer of Pt was deposited on top of the InGaAsP solar cell to prevent any damage to the device from Ga ion milling. The core-level spectra and valence-band spectra of the InGaAsP, InP and TiO_2 were characterized using an X-ray photoelectron spectrometer (Thermo ESCALAB250Xi) with a monochromatic Al K-alpha X-ray source (energy 1486.68 eV). The equipment was then operated in ultraviolet photoelectron spectroscopy (UPS) mode, using helium I (21.2 eV) as the UV source, to measure the work function of the InGaAsP, InP and TiO_2. Surface morphology of the perovskite layer was examined using field emission scanning electron microscopy (FE-SEM; FEI Verios) operated at an acceleration voltage of 5 kV and a current of 13 pA. High-resolution x-ray diffraction data were obtained using a PANalytical X'Pert PRO MRD system with Cu $K\alpha$ as the x-ray source. Photoluminescence measurements on InGaAsP samples were performed using a pulsed Yb:YAG laser with a wavelength of 522 nm. The excitation pulse had a duration of 300 fs and a repetition rate of 20.8 MHz. Reflectance/transmittance measurements of the devices were carried out using a PerkinElmer Lambda 1050 UV/vis/NIR spectrophotometer in an integrating sphere mode.

Device modelling

The simulations were performed using the COMSOL v6.1 semiconductor simulation module. Device simulations were performed in one dimension. The optical generation profile in the InGaAsP absorber layer was modelled using OPAL v2.0 from PV Lighthouse, using n and k data from Burkhard *et al*.[2] To simulate IQE profiles, we computed monochromatic generation profiles using Burkhard *et al.'s* optical data. Total generation was normalized across wavelengths and the collected photocurrent at each wavelength computed at short-circuit conditions. The properties of the different layers used in the simulations are tabulated below.

Table S1. The material characteristics of various layers within an InGaAsP heterojunction solar cell utilized during the simulation are as follows.

**Layer thicknesses**		
InGaAsP	1.5	µm
InP substrate	5	µm
TiO_2	10	nm
ITO	60	nm
**Material Properties**		
InGaAsP		
Direct recombination coefficient	10^{-10}	$cm^3\ s^{-1}$
Electron Affinity	4.21	eV
Bandgap	1.04	eV
Effective density of states, electrons and holes	$2x10^{19}$	cm^{-3}
Mobility, electrons	3500	$cm^2\ V^{-1}s^{-1}$
Mobility, holes	100	$cm^2\ V^{-1}s^{-1}$
Bulk doping	nil (intrinsic)	cm^{-3}
TiO_2		
Electron affinity	4.0	eV
Bandgap	3.2	eV
Bulk doping (n-type)	10^{19}	cm^{-3}
Mobility, electrons	0.1	$cm^2\ V^{-1}s^{-1}$
Mobility, holes	2	$cm^2\ V^{-1}s^{-1}$
InP		
Electron affinity	4.2	eV
Bandgap	1.34	eV
Electron mobility	5400	$cm^2\ V^{-1}s^{-1}$
Hole mobility	200	$cm^2\ V^{-1}s^{-1}$
Doping (p-type)	$5x10^{18}$	cm^{-3}
ITO		
Electron affinity	4.2	eV
Bandgap	4.1	eV

Electron mobility	5	$cm^2 V^{-1}s^{-1}$
Hole mobility	1	$cm^2 V^{-1}s^{-1}$
Doping (n-type)	10^{20}	cm^{-3}

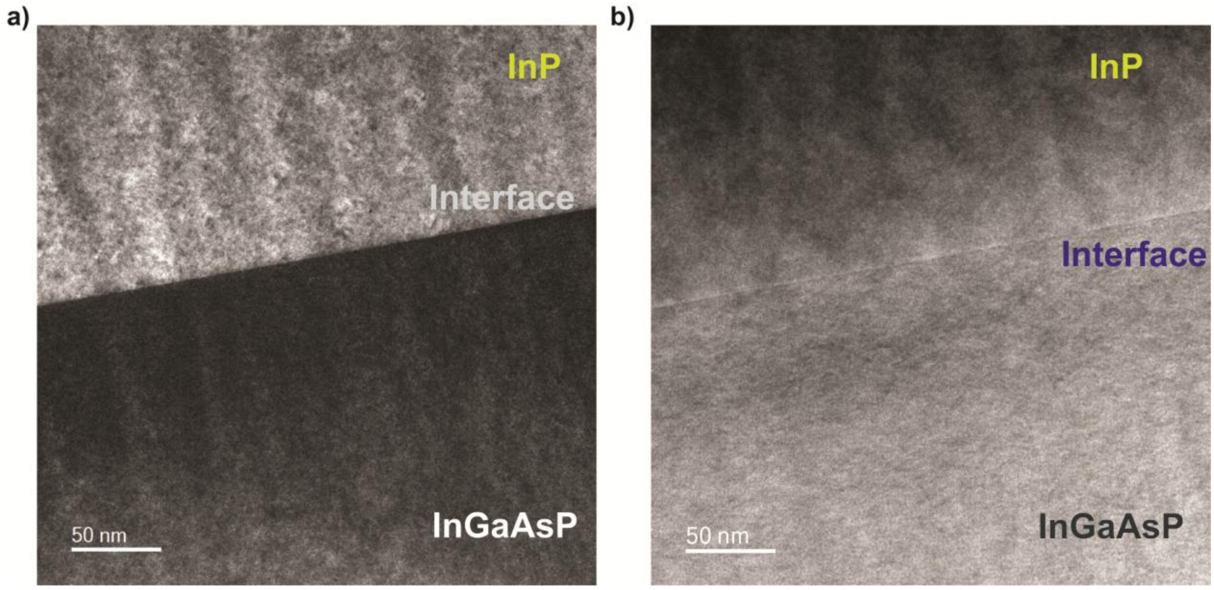

Figure S1. Transmission electron micrographs of the InGaAsP/InP structure using (a) bright field imaging and (b) dark field imaging. The images show excellent lattice matching condition between the InGaAsP layer and the InP wafer.

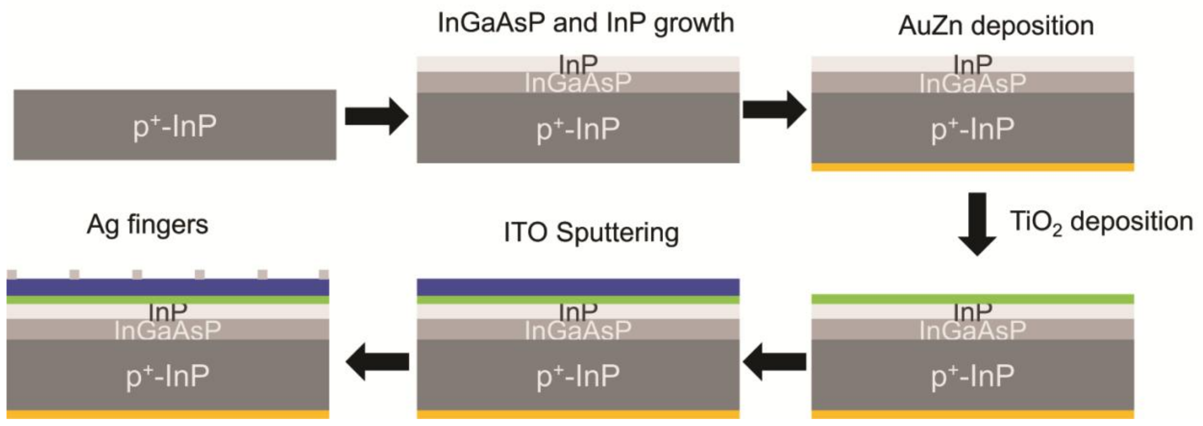

Figure S2. Schematic illustration of InGaAsP solar cell fabrication process flow.

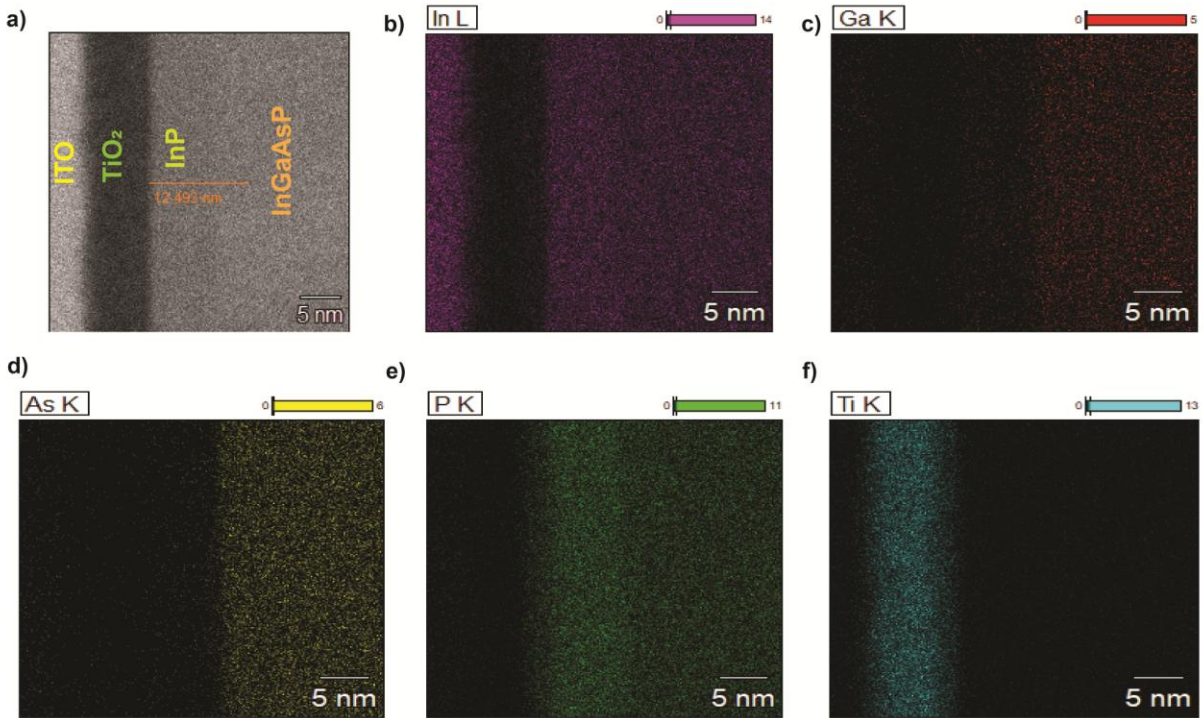

Figure S3. Cross-sectional elemental mapping of InGaAsP solar cell acquired using transmission electron microscopy.

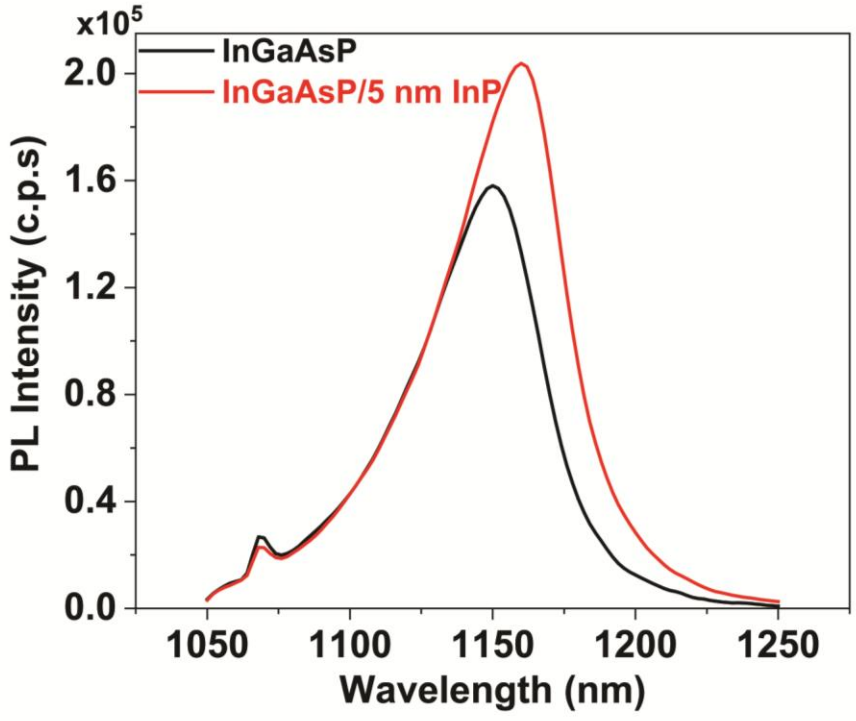

Figure S4. Photoluminescence spectra demonstrating the impact of InP passivation layer on InGaAsP photoabsorber material.

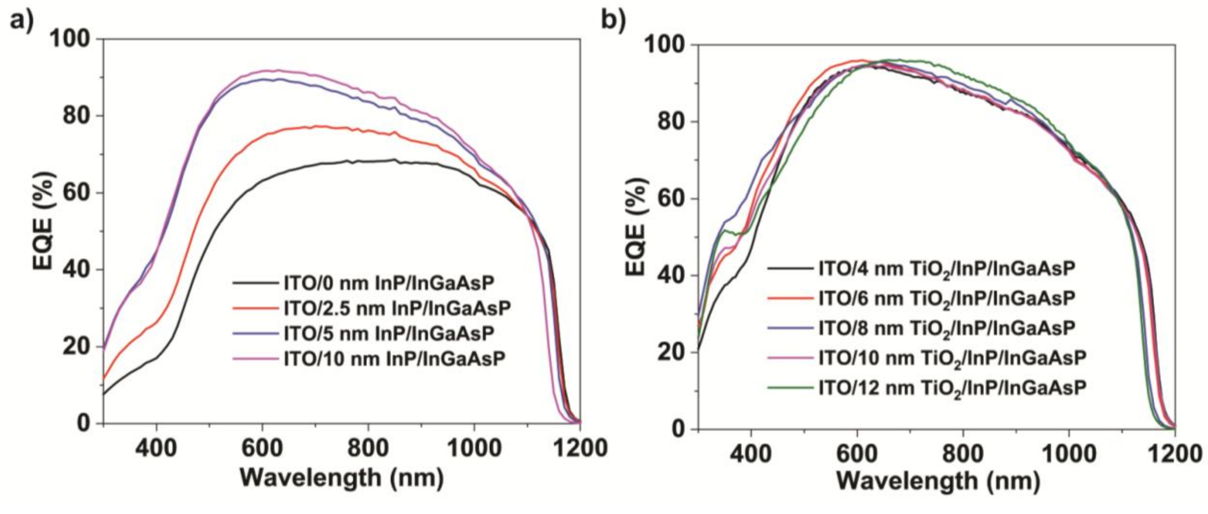

Figure S5. External quantum efficiencies of InGaAsP solar cell (a) with different thicknesses of InP passivation layer and (b) different thicknesses of TiO_2 ESC layer with 10 nm-thick InP passivation layer.

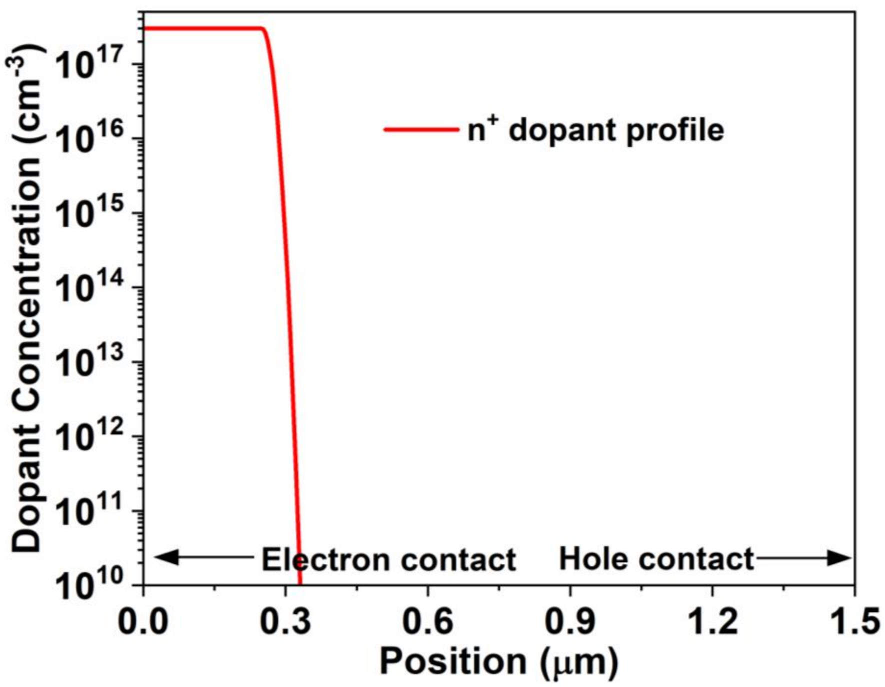

Figure S6. Plot of n^+ surface dopant profile used in the device simulations.

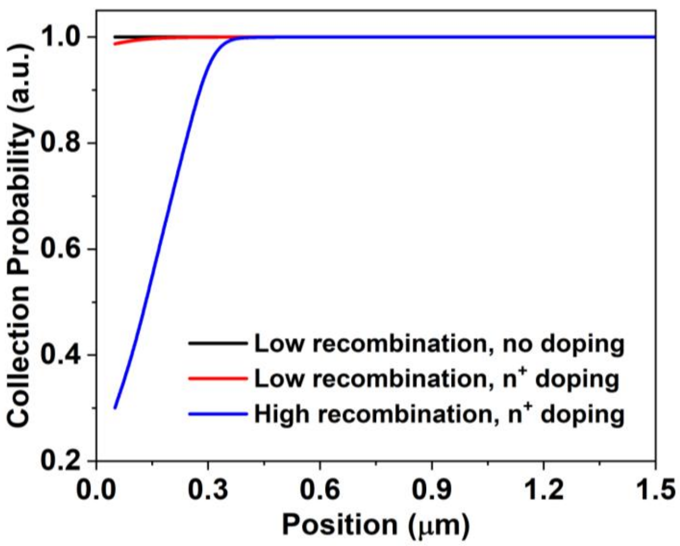

Figure S7. Charge collection probability as a function of position in the 1.5 μm-thick InGaAsP absorber layer modelled in this work.

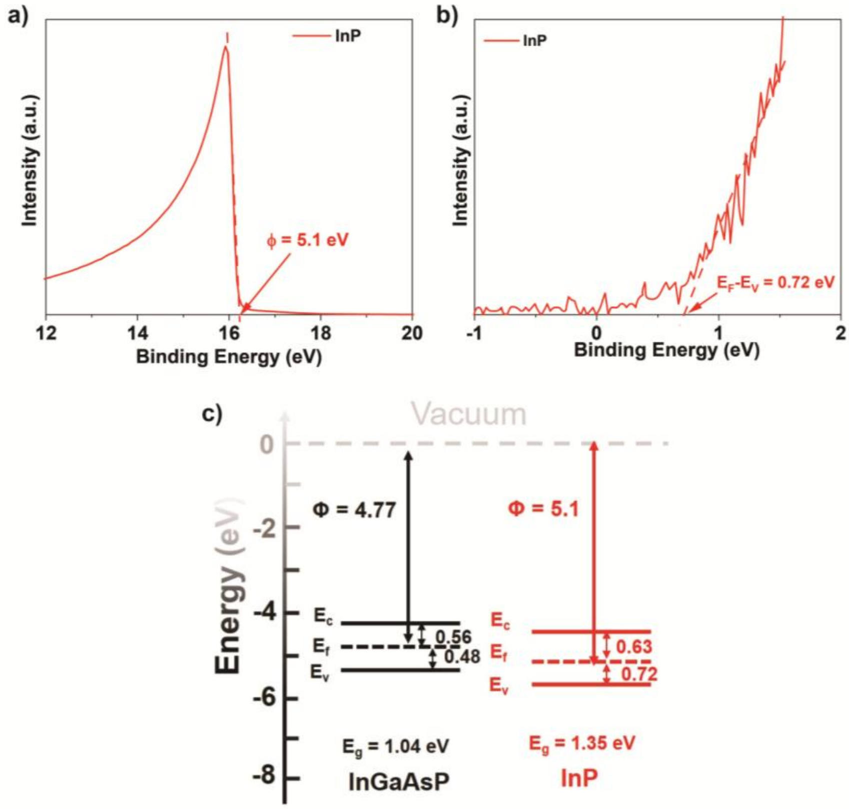

Figure S8. X-ray and ultraviolet photoelectron spectroscopy of InP and InGaAsP. (a) Secondary electron cutoff spectrum of InP and InGaAsP. (b). Valence band spectrum of InP and InGaAsP (c) Schematic of the band diagram at the InP/InGaAsP interface derived from XPS and UPS measurements.

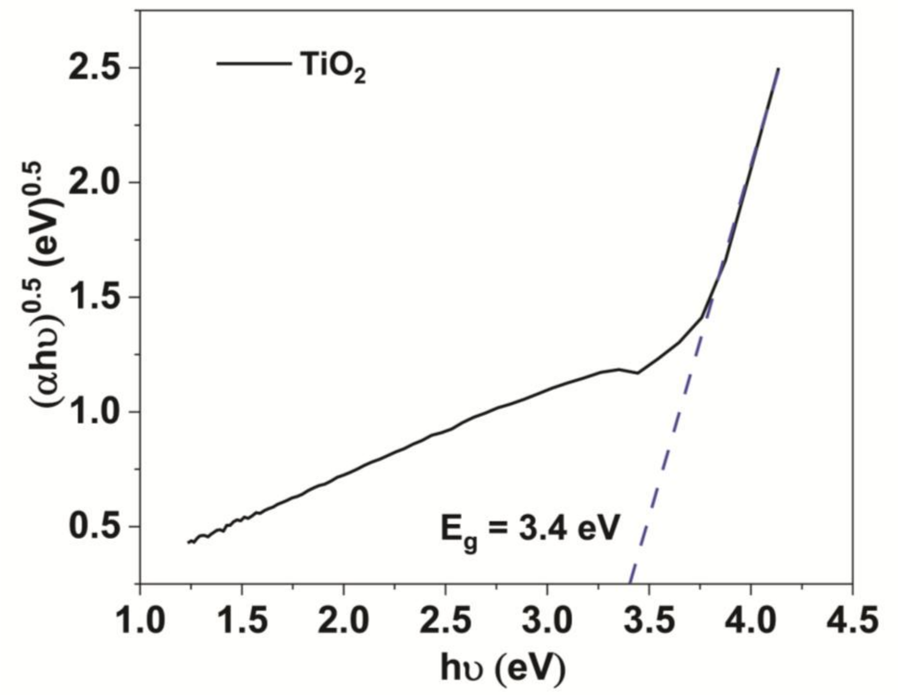

Figure S9. Tauc plot of TiO_2 ESC layer to derive its optical bandgap.

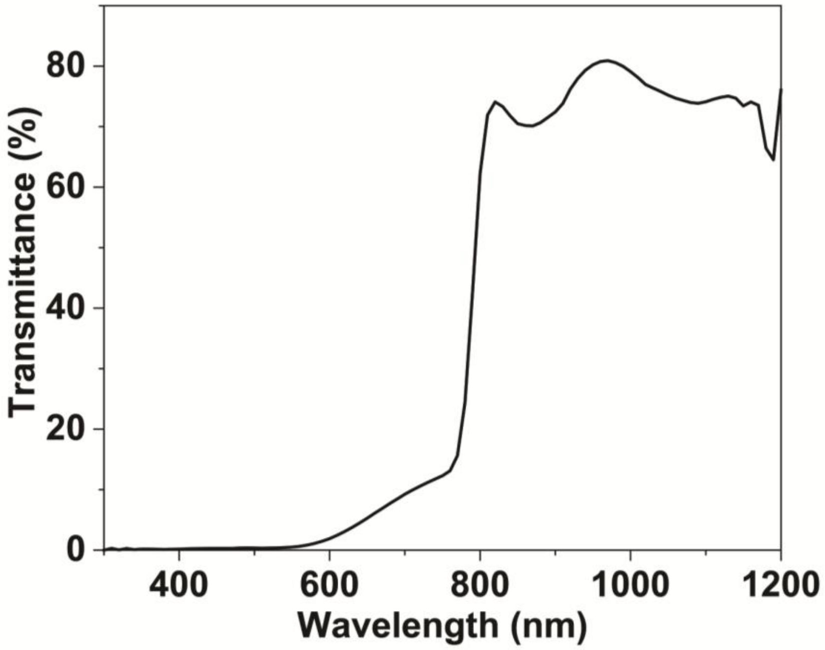

Figure S10. UV-Vis transmittance curve of semi-transparent perovskite solar cell.

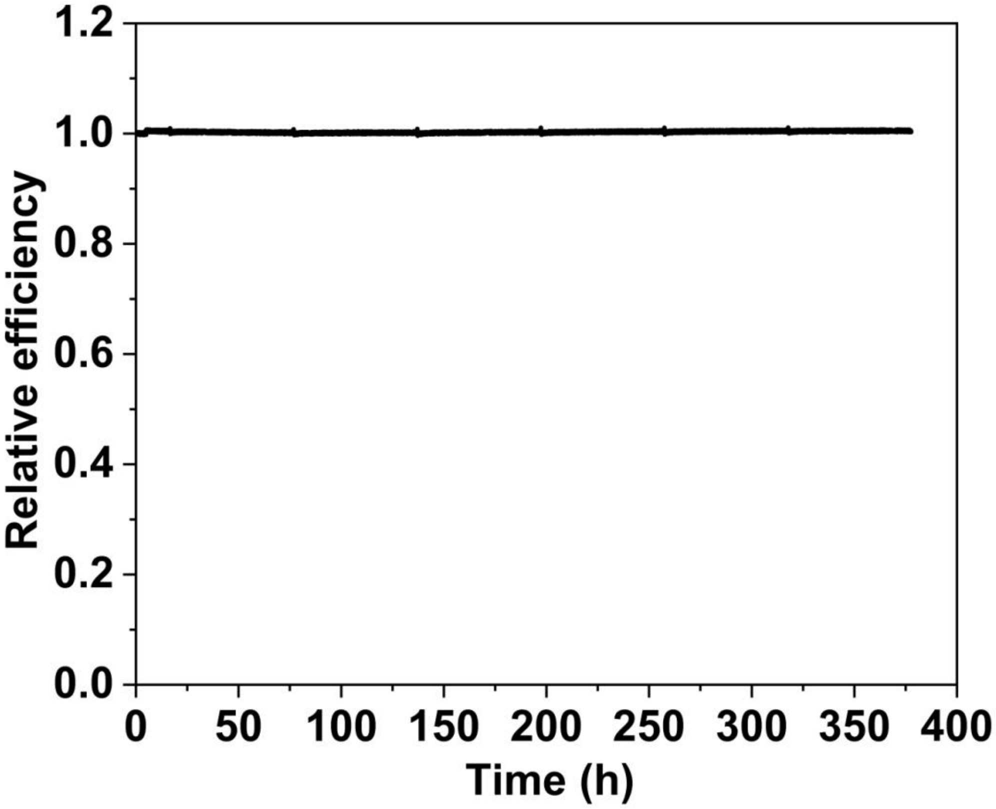

Figure S11. Perovskite solar cell stability data.

Table S2. Comparison of open circuit voltage (V_{oc}) calculated from the measurements performed in dark and under 1 sum simulated illumination with different thickness of InP passivation layer.

**Devices**	**V_{oc} (1 sun condition)**	**V_{oc} (dark)**
ITO/0 nm InP/InGaAsP	522 mV	542 mV
ITO/2.5 nm InP/InGaAsP	513 mV	529 mV
ITO/5 nm InP/InGaAsP	552 mV	568 mV
ITO/10 nm InP/InGaAsP	596 mV	596 mV
ITO/10 nm InP/InGaAsP	593 mV	576 mV

Table S3. Comparison of solar cell performance of various InGaAsP solar cells reported in literature.

**Device Architecture**	**Bandgap (eV)**	**V_{oc} (mV)**	**J_{sc} (mA cm^{-2})**	**FF (%)**	**η (%)**	**Ref.**
InGaAsP homojunction	1.61-1.65	1002	11.9	80.7	9.6	[3]
InGaAsP homojunction	1	500	35.2	76.8	13.2	[4]
InGaAsP homojunction	1.05	590	26.3	78.1	12.1	[5]
InGaAsP homojunction	1	545	44.5	77.3	18.8	[6]
InGaAsP homojunction	1	517	41.5	75.3	16.2	[7]
InGaAsP homojunction	1	693	36.1	76.1	19.1	[8]
InGaAsP homojunction	1.04	540	27.6	79.7	11.9	[9]
ITO/InGaAsP	1.55	560	28.8	67.7	10.9	[10]
ITO/InGaAsP	1.55	600	24.5	65.3	9.6	[11]
InGaAsP homojunction	1.7	1130	19.2	85.4	18.7	[12]
**TiO_2/InP/InGaAsP heterojunction**	**1.04**	**657**	**40.4**	**71.8**	**19.0**	**This work**

Table S4 Table comparing the *Jsc* data obtained from *JV* curve and integration of EQE.

Devices	*Jsc* (from *JV* curve) (mA cm^{-2})	*Jsc* (From EQE) (mA cm^{-2})	% difference
ITO/InGaAsP	30.1	28.8	2.3
ITO/TiO_2/InGaAsP	38.6	36.9	4.4
ITO/ InP/InGaAsP	36.4	35.1	3.5
ITO/TiO_2/InP/InGaAsP	40.4	38.6	4.5

Photovoltaic Power and Fill Factor Loss Analysis at maximum power point:

The loss calculation performed in this paper is based on the work of Aberle *et al*.[13] For loss calculation, we made a precise measurement of following physical parameters:

(1) 1-sun *J-V* curve: The precise measurement of light *J-V* of the proposed heterojunction solar cell was done using an Oriel solar simulator. Before every measurement the solar simulator was calibrated to one sun using a standard sample provided by the company. The one sun *J-V* curve was evaluated to obtain the V_{oc}, J_{sc}, *FF*, and efficiency of the device.

(2) Dark *J-V*: Dark *J-V* measurement of the samples were made using the same simulator. However, for precise dark *J-V*, the sample was covered with a thick aluminum foil and the lights were switched off before performing the measurements.

(3) Light series and shunt resistances were extracted by fitting the light *J-V* curve using double diode equation.

Equation used for loss analysis are:

$$Shading\ fraction = \frac{Fingers\ area}{Device\ area} \times 100$$
#S1

Where fingers area is the area covered by metal fingers and device area is the masked area of the solar cell.

$$Metal\ shading\ loss = Shading\ fraction\ \times Vmpp\ \times Jmpp$$
#S2

$$J_{sc(EQE)} = \int_{\lambda 2}^{\lambda 1} \frac{q\lambda}{hc} \{EQE\ (\lambda). AM\ 1.5G\ (\lambda)\} d\lambda$$
#S4

$$Series\ resistance\ loss = R_{s(light)} \times J_{mpp}^2$$
#S5

$$Shunt\ resistance\ loss = \frac{V_{shunt}^2}{R_{shunt}}$$ #S6

$$V_{shunt} = V_{mpp} + \left(R_{s\ (light)} \times J_{mpp}^2\right)$$ #S7

where J_{mpp} and V_{mpp} are the current density and voltage at MPP operation respectively and R_s and R_{sh} are the series resistance and shunt resistance of the solar cell under illumination.

The current loss at maximum power point can be written as:

$$Current\ loss\ at\ MPP = J_{sc} - J_{mpp} - J_{shunt}$$
#S8

$$J_{shunt} = \frac{V_{shunt}}{R_{shunt}}$$
#S9

In the above equation, is the J_{mpp} recombination current at maximum power point, whereas, J_{shunt} is current loss due to shunt. Therefore, the current loss at MPP is due to recombination in different regions of the solar cells at maximum power point.

In addition, loss due to non-perfect IQE was calculated assuming that under ideal condition, IQE should be 100% within 300-920 nm.

Table S5. Power and fill factor loss analysis at the maximum power point (V_{mpp}).

S.N.	Losses	Power loss (mW cm^{-2})	Description
**1**	Metal shading	0.5	fractional loss of J_{mpp} based on the metal shading area
**2**	Front surface reflectance (includes front surface escape)	2.4	difference between IQE- and EQE-based currents
3	**Total optical losses (300−1200 nm)**	**2.9**	**Sum of 1 and 2**
**4**	Series resistance loss	0.74	$(J_{mpp})^2 \times R_s$
**5**	Shunt resistance loss	0.00	$(V_{shunt})/R_{sh}$, $V_{shunt} = (V_{mpp} + J_{mpp} \times R_s)$
6	**Total resistance loss**	**0.74**	**Sum of 4 and 5**
**7**	Non-perfect IQE	2.3	difference between QE = 100% and IQE-based current
**8**	Forward-bias current loss at MPP	2.5	difference between J_{sc} and J_{mpp},
9	**Total recombination losses**	**4.8**	**Sum of 7 and 8**
**10**	**Total losses**	**8.4**	**Sum of 3, 6 and 9**
**11**	**Total efficiency potential of the solar cell**	**27.4**	**maximum possible efficiency of the current solar cell**
	**fill factor loss (*FF*) analysis**		
	**J_{01} limit of the *FF* (FF_0) (%)**		**83.9**
	measured *FF* (%)		71.8
	absolute *FF* loss due to R_s (%)		5.7
	absolute *FF* loss due to R_{sh} (%)		0.1
	absolute *FF* loss due to J_{02} diode recombination component (%)		6.3
	**total *FF* loss (%)**		**12.1**

References:

[1] T. Duong, T. Nguyen, K. Huang, H. Pham, S. G. Adhikari, M. R. Khan, L. Duan, W. Liang, K. C. Fong, H. Shen, *Advanced Energy Materials* **2023**, 13, 2203607.

[2] H. Burkhard, H. Dinges, E. Kuphal, *Journal of Applied Physics* **1982**, 53, 655.

[3] T. Sugaya, Y. Nagato, Y. Okano, R. Oshima, T. Tayagaki, K. Makita, K. Matsubara, *Journal of Vacuum Science & Technology B* **2017**, 35.

[4] L. Ji, M. Tan, C. Ding, K. Honda, R. Harasawa, Y. Yasue, Y. Wu, P. Dai, A. Tackeuchi, L. Bian, *Journal of Crystal Growth* **2017**, 458, 110.

[5] R. Oshima, K. Makita, T. Tayagaki, T. Sugaya, presented at 2016 IEEE 43rd Photovoltaic Specialists Conference (PVSC) **2016**.

[6] L. Ji, M. Tan, K. Honda, R. Harasawa, Y. Yasue, Y. Wu, P. Dai, A. Tackeuchi, L. Bian, S. Lu, *Solar Energy Materials & Solar Cells* **2015**, 137, 68.

[7] W.-x. Yang, P. Dai, L. Ji, M. Tan, Y.-y. Wu, S. Uchida, S.-l. Lu, H. Yang, *Applied Surface Science* **2016**, 389, 673.

[8] H. Lu, X. Li, W. Zhang, G. Li, S. Hu, N. Dai, *Solar Energy Materials & Solar Cells* **2019**, 196, 65.

[9] S. Park, M. R. McCartney, D. J. Smith, J. Jeon, Y. Kim, S. J. Lee, *Journal of Materials Chemistry A* **2022**, 10, 13106.

[10] S. Matsubara, H. Narui, N. Tsuchiya, N. S. i. Takahashi, S. Kurita, *Journal of applied physics* **1989**, 66, 3337.

[11] H. Narui, S. Matsubara, N. S.-i. Takahashi, S. Kurita, *Japanese journal of applied physics* **1987**, 26, L91.

[12] N. Jain, J. Simon, K. L. Schulte, D. J. Friedman, D. R. Diercks, C. E. Packard, D. L. Young, A. J. Ptak, *IEEE Journal of Photovoltaics* **2018**, 8, 1577.

[13] A. G. Aberle, W. Zhang, B. Hoex, *Energy Procedia* **2011**, 8, 244.
